# Comparison of the efficacy and safety of different thrombolytic drugs in the treatment of acute ischemic stroke within 4.5 h: a systematic review and network meta-analysis

**DOI:** 10.3389/fneur.2026.1775325

**Published:** 2026-03-27

**Authors:** Xinmin Yu, Jiale Liu, Chenlei Shi, Degang Xue, Yuting Jiang, Weiwei Wang, Miaomiao Zhao

**Affiliations:** 1 Graduate School, Shanghai University of Traditional Chinese Medicine, Shanghai, China; 2 School of Clinical Medicine, Shanghai University of Medicine and Health Sciences, Shanghai, China; 3 Department of Cardiology, General Hospital of Fushun Mining Bureau of Liaoning Health Industry, Fushun, Liaoning, China; 4 Department of Public Health, Jinshan Central Hospital Affiliated to Shanghai University of Medicine and Health Sciences, Shanghai, China; 5 Shanghai University of Traditional Chinese Medicine, Shanghai, China

**Keywords:** acute ischemic stroke, efficacy, network meta-analysis, safety, thrombolytics, within 4.5 h onset

## Abstract

**Background:**

Although several newer thrombolytic agents for acute ischemic stroke (AIS) within 4.5 h of onset have been developed, their relative efficacy, safety, and optimal dosing remain unclear. A comprehensive comparison across all available agents is therefore needed.

**Methods:**

We systematically searched PubMed, Embase, the Cochrane Central Register of Controlled Trials, and Web of Science for English-language reports of randomized controlled trials (RCTs) published up to December 12, 2025. Eligible trials enrolled adult AIS patients treated with intravenous thrombolysis. Primary outcomes were 90-day excellent (modified Rankin Scale [mRS] score 0–1) and good (mRS 0–2) functional outcomes. Safety outcomes were symptomatic intracranial hemorrhage (sICH), and all-cause mortality. A frequentist network meta-analysis using a fixed-effect consistency model was conducted to estimate odds ratios (ORs) with 95% confidence intervals (CIs).

**Results:**

A total of 21 RCTs involving 16,837 patients were included. For achieving mRS 0–1, reteplase (18 + 18 mg) showed a statistically significant advantage over alteplase (0.9 mg/kg) (OR 1.60; 95% CI, 1.27–2.02), and non-immunogenic recombinant staphylokinase (10 mg) demonstrated a statistically significant benefit (OR 2.23; 95% CI, 1.43–3.48). Reteplase (18 + 18 mg) also improved the likelihood of mRS 0–2 compared with alteplase (OR 1.41; 95% CI, 1.08–1.84). For safety, non-immunogenic recombinant staphylokinase significantly reduced sICH risk compared with tenecteplase 0.25 mg/kg (OR 0.31; 95% CI, 0.11–0.94). No significant differences in 90-day mortality were observed among treatments.

**Conclusion:**

Within the 4.5-h treatment window, reteplase (18 + 18 mg) and non-immunogenic recombinant staphylokinase (10 mg) were associated with the highest probabilities of improved functional outcomes.

**Systematic review registration:**

https://www.crd.york.ac.uk/PROSPERO/view/CRD420251152754.

## 1 Introduction

Acute ischemic stroke (AIS) remains one of the leading causes of death and long-term disability worldwide, imposing a substantial clinical and economic burden. In 2021, ischemic stroke accounted for approximately 65% of all stroke cases, resulting in 7.8 million new events, 3.59 million deaths, and more than 70 million disability-adjusted life years (1, 2). The global economic impact of stroke is estimated to exceed $890 billion annually (3). These figures underscore the urgent need for effective therapeutic strategies that can improve functional outcomes and reduce mortality.

Timely restoration of cerebral blood flow within the 4.5-h therapeutic window is critical for improving outcomes in AIS (4, 5). Intravenous thrombolysis with alteplase remains the guideline-endorsed first-line thrombolytic agent during this period (6, 7); however, its clinical utility is limited by a substantial risk of symptomatic intracranial hemorrhage (sICH) (8) and by the need for continuous intravenous infusion, which may delay treatment in time-sensitive emergency settings (9).

To overcome these limitations, attention has shifted toward thrombolytic agents with more favorable pharmacological properties. Tenecteplase, a genetically modified variant of alteplase, offers higher fibrin specificity and a longer half-life, enabling single-bolus administration (10, 11). However, evidence regarding its superiority over alteplase remains inconclusive, and its optimal dosing (e.g., 0.25 vs. 0.40 mg/kg) continues to be debated (12). Other emerging agents-including recombinant human prourokinase (rhPro-UK) and non-immunogenic recombinant staphylokinase-have shown promise but lack robust comparative data from large-scale randomized trials, resulting in substantial uncertainty regarding their relative efficacy and safety.

Although numerous randomized controlled trials (RCTs) have compared these newer agents with alteplase, their findings are often inconsistent due to small sample sizes, heterogeneous patient populations, and variation in outcome definitions. Such fragmentation of evidence complicates informed clinical decision-making. Conventional pairwise meta-analyses cannot address these limitations because they are restricted to direct comparisons and cannot simultaneously evaluate multiple interventions in the absence of head-to-head trials.

Network meta-analysis (NMA) allows the integration of direct and indirect evidence, enabling simultaneous comparison and ranking of multiple thrombolytic strategies. Accordingly, we conducted a comprehensive NMA, accompanied by a CINeMA-based confidence assessment, to evaluate and compare the efficacy and safety of available thrombolytic regimens for AIS within 4.5 h of onset. Our objective was to provide evidence-based treatment rankings that may inform future clinical practice and guideline development.

## 2 Methods

We conducted a systematic review and frequentist NMA of RCTs in accordance with the PRISMA 2020 guidelines. The study protocol was prospectively registered in PROSPERO (CRD420251152754).

### 2.1 Data sources and search strategies

A comprehensive search of PubMed, Embase, Web of Science, and the Cochrane Central Register of Controlled Trials was conducted from database inception to 12 December 2025. The search was restricted to English-language publications. Two reviewers independently performed the search and study selection, with disagreements resolved through discussion. The search strategy incorporated the following terms:

((Stroke OR Ischemic stroke) AND (“Alteplase” OR “Tenecteplase” OR “Reteplase” OR “Urokinase” OR “Desmoteplase” OR “rt-PA” OR “recombinant tissue plasminogen activator” OR “TNK”)) AND (“randomized controlled trial” OR “randomised controlled trial” OR “rct” OR “random allocation” OR “randomly allocated” OR random*)

### 2.2 Inclusion and exclusion criteria

Eligible studies included RCTs enrolling adults (≥18 years) with neuroimaging-confirmed AIS treated within 4.5 h of symptom onset. Trials evaluating intravenous thrombolytic agents were included, whereas studies incorporating endovascular thrombectomy were excluded to avoid treatment-related confounding.

### 2.3 Outcomes

The interventions examined in this NMA included the following dosing regimens: alteplase (0.6 mg/kg and 0.9 mg/kg), tenecteplase (0.1 mg/kg, 0.25 mg/kg, 0.32 mg/kg, and 0.4 mg/kg), reteplase (12 mg + 12 mg and 18 mg + 18 mg), rhPro-UK (35 mg and 50 mg), non-immunogenic recombinant staphylokinase (10 mg), and placebo. Primary efficacy outcomes were 90-day excellent (modified Rankin Scale [mRS] score 0–1) and good (mRS 0–2) functional outcomes. Safety outcomes included 90-day all-cause mortality and sICH, which was defined according to criteria used in the individual trials.

The diagnostic criteria for sICH varied across key trials, including European Cooperative Acute Stroke Study III definition (ECASS III) (13–25), European Cooperative Acute Stroke Study II definition (ECASS II) (26, 27), Thrombolysis in Stroke-Monitoring Study criteria (SITS-MOST) (28, 29), and investigator-assessed criteria (30–33).

### 2.4 Study sections and data extraction

After removing duplicates using EndNote 20, two reviewers independently screened titles and abstracts, followed by full-text assessment of potentially eligible studies. Disagreements were resolved through consensus. Reasons for exclusion were documented, and the study selection process was presented in a PRISMA flow diagram.

Two reviewers independently extracted study characteristics, participant demographics, intervention details, and outcome data using a standardized form.

### 2.5 Quality assessment

The risk of bias in RCTs was assessed using the Cochrane Risk of Bias tool (34), in the following seven respects: (1) random sequence generation, (2) allocation concealment, (3) blinding of participants and personnel, (4) blinding of outcome assessment; (5) incomplete outcome data, (6) selective reporting, (7) other bias. Disagreements were resolved by a third reviewer. Publication bias and small-study effects were evaluated using funnel plots and Egger’ s test. Certainty of evidence was assessed using the CINeMA framework across six domains (35, 36).

### 2.6 Statistical analysis

A frequentist NMA was performed using the Stata MP 18.0 “network” suite. Odds ratios (ORs) with 95% confidence intervals (CIs) were calculated for all dichotomous outcomes.

Between-study heterogeneity was assessed using the I^2^ statistic, following the guidance outlined in the Cochrane Handbook for Systematic Reviews (37). An I^2^ value of 0–50% was considered indicative of low heterogeneity, 50–75% of moderate heterogeneity, and 75–90% of high heterogeneity (37). A fixed-effect model was employed when heterogeneity was low (I^2^ < 50% and *p* > 0.05 for the Cochran’s Q test); otherwise, a random-effect model was used (38–40). Network consistency was evaluated globally using the consistency testing. In the absence of significant inconsistency, a fixed-effect consistency model was adopted.

Transitivity represents the fundamental assumption for conducting an NMA. Therefore, we compared the distributions of clinical variables (e.g., baseline age, course of disease, and sample size) that may serve as effect modifiers across treatment comparisons to assess transitivity and potential for inclusion in NMA.

Treatment rankings were estimated using the surface under the cumulative ranking curve (SUCRA) (41), where higher SUCRA values reflect a greater likelihood of superior efficacy for outcomes such as an mRS score of 0–1, and a higher probability of increased risk for safety endpoints like sICH. League tables were generated to display relative treatment effects. Publication bias and small-study effects were explored using funnel plots and Egger’s test when ≥10 studies were available.

Sensitivity analyses were conducted by applying alternative statistical models to evaluate the consistency of the primary results. Subgroup analyses were conducted for each distinct dose regimen of rhPro-UK, tenecteplase, and reteplase, with each dose modeled as a separate node and compared directly against alteplase 0.9 mg/kg. Statistical significance was defined as a two-sided *p* < 0.05 for all analyses.

## 3 Results

### 3.1 Study selection and characteristics

**Figure 1:**
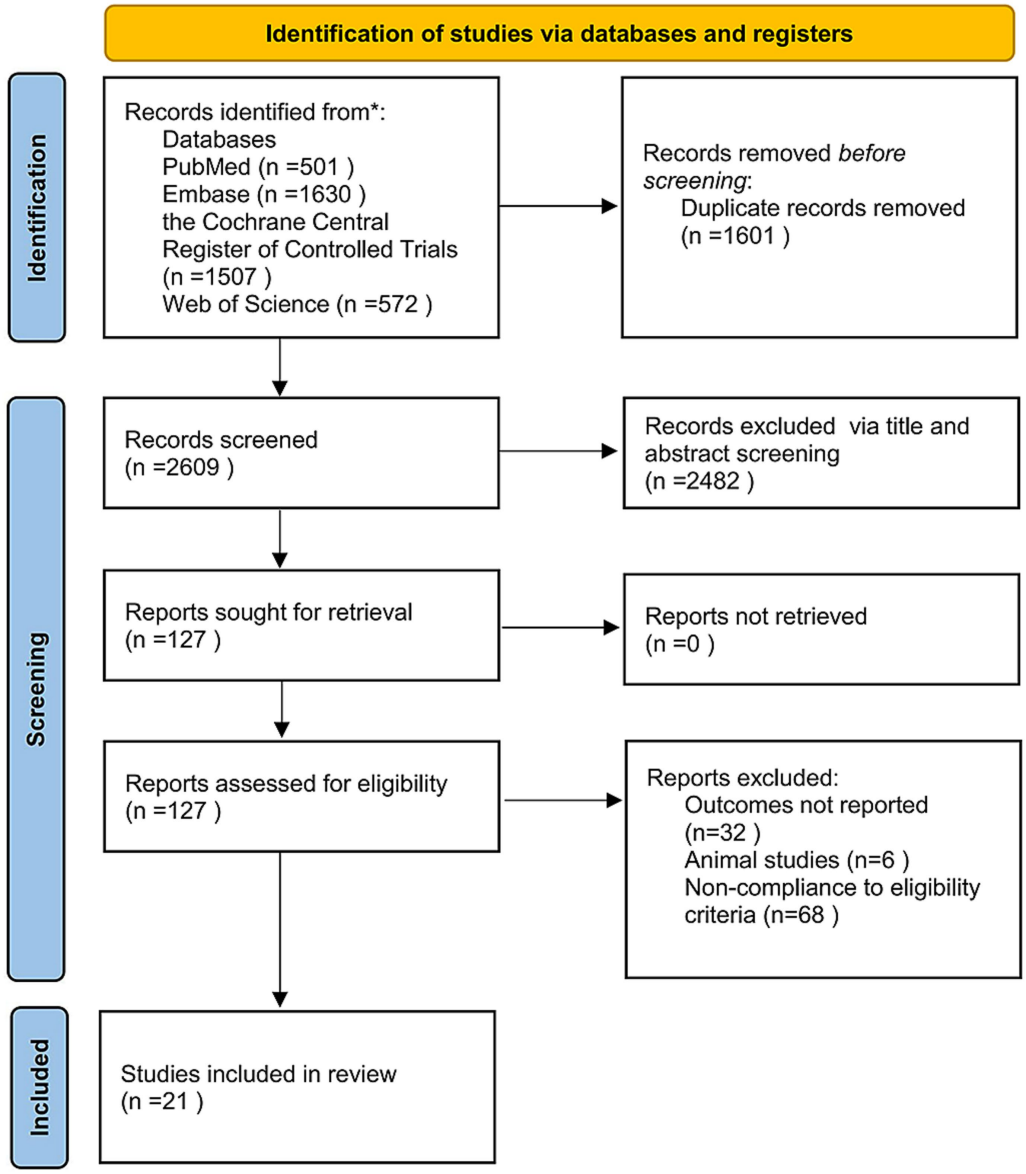
PRISMA flow chart.

A total of 4,210 unique records were retrieved through the initial literature search. After title and abstract screening, 2,609 articles were selected for full-text review, and 21 RCTs ultimately met the inclusion criteria (Figure 1). Key characteristics of these studies are summarized in Table 1.

**Table 1:** Characteristics of included studies.

Author, Year	Course of disease	Intervention	Control	Population			NIHSS, median		Age	Outcomes
				All (men, %)	Intervention	Control	Intervention	Control		
Xia Meng, 2024 (19)	4.5 h	Tenecteplase 0.25 mg/kg	Alteplase 0.9 mg/kg	1,465 (1,019, 69.6)	732	733	6	6	65.5	ABCD
Yongjun Wang, 2023 (25)	4.5 h	Tenecteplase 0.25 mg/kg	Alteplase 0.9 mg/kg	1,417 (971, 68.5)	710	707	7	7	66.0	ABCD
Shuya Li, 2024 (17)	4.5 h	Reteplase 18 + 18 mg	Alteplase 0.9 mg/kg	1,412 (996, 70.5)	707	705	6	6	63.0	ABCD
Werner Hacke, 2008 (14)	3–4.5 h	Alteplase 0.9 mg/kg	Placebo	821 (515, 62.7)	418	403	9	10	68.0	ABCD
Xuya Huang, 2015 (27)	4.5 h	Tenecteplase 0.25 mg/kg	Alteplase 0.9 mg/kg	104 (61, 58.7)	47	49	12	11	71.0	ABCD
Keith W Muir, 2024 (20)	4.5 h	Tenecteplase 0.25 mg/kg	Alteplase 0.9 mg/kg	1777 (1,060, 59.7)	885	892	NA	NA	70.4	ABCD
Bruce C V Campbell, 2020 (32)	4.5 h	Tenecteplase 0.40 mg/kg	Tenecteplase 0.25 mg/kg	300 (159, 53.0)	150	150	17	16	72.8	ABCD
Mark W Parsons, 2024 (21)	4.5 h	Tenecteplase 0.25 mg/kg	Alteplase 0.9 mg/kg	680 (420, 61.8)	339	341	NA	NA	74.0	ABCD
Christopher Elnan Kvistad, 2017 (15)	4.5 h	Tenecteplase 0.25 mg/kg	Alteplase 0.9 mg/kg	204 (98, 48.0)	100	104	NA	NA	70.9	ABCD
Andrew Bivard, 2022 (28)	4.5 h	Alteplase 0.9 mg/kg	Tenecteplase 0.25 mg/kg	104 (63, 60.6)	49	55	8	8	74.5	ABCD
Shuya Li, 2025 (16)	4.5 h	rhPro-UK 35 mg	Alteplase 0.9 mg/kg	1,552 (1,032, 66.5)	775	777	7	7	65.0	ABCD
Haiqing Song, 2022 (23)	4.5 h	rhPro-UK 35/50 mg	Alteplase 0.9 mg/kg	112 (73, 65.2)	rhPro-UK 35 mg: 36 rhPro-UK 50 mg: 38	38	NA	NA	60.5	ABCD
Haiqing Song, 2023 (24)	4.5 h	rhPro-UK 35 mg	Alteplase 0.9 mg/kg	663 (502, 75.7)	330	333	6	6	61.0	ABCD
Eugene I Gusev, 2021 (13)	4.5 h	Staphylokinase 10 mg	Alteplase 0.9 mg/kg	336 (218, 64.9)	168	168	11	11	64.5	ABCD
Shuya Li, 2024 (18)	4.5 h	Tenecteplase 0.1/0.25/0.32 mg/kg	Alteplase 0.9 mg/kg	236 (170, 72.0)	Tenecteplase 0.1 mg/kg: 60 Tenecteplase 0.25 mg/kg: 57 Tenecteplase 0.32 mg/kg: 60	59	Tenecteplase 0.1 mg/kg: 7 Tenecteplase 0.25 mg/kg: 8 Tenecteplase 0.32 mg/kg: 7.5	8	64.5	ABCD
Shuya Li, 2023 (26)	4.5 h	Reteplase 12 + 12/18 + 18 mg	Alteplase 0.9 mg/kg	180 (130, 72.2)	Reteplase 12 + 12 mg: 60 Reteplase 18 + 18 mg: 66	50	Reteplase 12 + 12 mg: 6 Reteplase 18 + 18 mg: 6	8	62.7	ABCD
Craig S Aanderson, 2016 (29)	4.5 h	Alteplase 0.6 mg/kg	Alteplase 0.9 mg/kg	3,297 (2049, 62.1)	1,654	1,643	8	8	67.5	ABCD
Nishita Singh, 2023 (30)	4.5 h	Tenecteplase 0.25 mg/kg	Alteplase 0.9 mg/kg	1,538 (803, 52.2)	787	751	NA	NA	73.5	ABCD
Ole Morten Rønning, 2019 (22)	3–4.5 h	Tenecteplase 0.40 mg/kg	Alteplase 0.9 mg/kg	194 (113, 58.2)	105	89	3	3	66.2	ACD
Study Group,1995 (33)	0-3 h	Alteplase 0.9 mg/kg	Placebo	333 (248, 74.5)	168	165	14	15	67.5	ABCD
E Clarke Haley, 2010 (31)	0-3 h	Tenecteplase 0.1/0.25/0.40 mg/kg	Alteplase 0.9 mg/kg	112 (58, 51.8)	Tenecteplase 0.1 mg/kg: 31 Tenecteplase 0.25 mg/kg: 31 Tenecteplase 0.40 mg/kg: 19	31	Tenecteplase 0.1 mg/kg: 8 Tenecteplase 0.25 mg/kg: 10 Tenecteplase 0.40 mg/kg: 9	13	69.0	ACD

A, excellent functional outcome; B, good functional outcome; C, 90-day all-cause mortality; D, symptomatic intracranial hemorrhage events.

Across the 21 RCTs, 16,837 patients with AIS treated within 4.5 h of symptom onset were included after exclusion of those lost to follow-up or who discontinued participation. Patients received one of five intravenous thrombolytic agents-alteplase, tenecteplase, reteplase, rhPro-UK, or non-immunogenic recombinant staphylokinase or placebo. Eleven dosing regimens were evaluated: alteplase (0.6 mg/kg, 0.9 mg/kg), tenecteplase (0.1 mg/kg, 0.25 mg/kg, 0.32 mg/kg, 0.4 mg/kg), reteplase (12 + 12 mg, 18 + 18 mg), rhPro-UK (35 mg, 50 mg), and non-immunogenic staphylokinase (10 mg). Placebo administration varied across trials. Detailed dosing schemes and administration approaches are presented in Supplementary Table 1.

**Figure 2:**
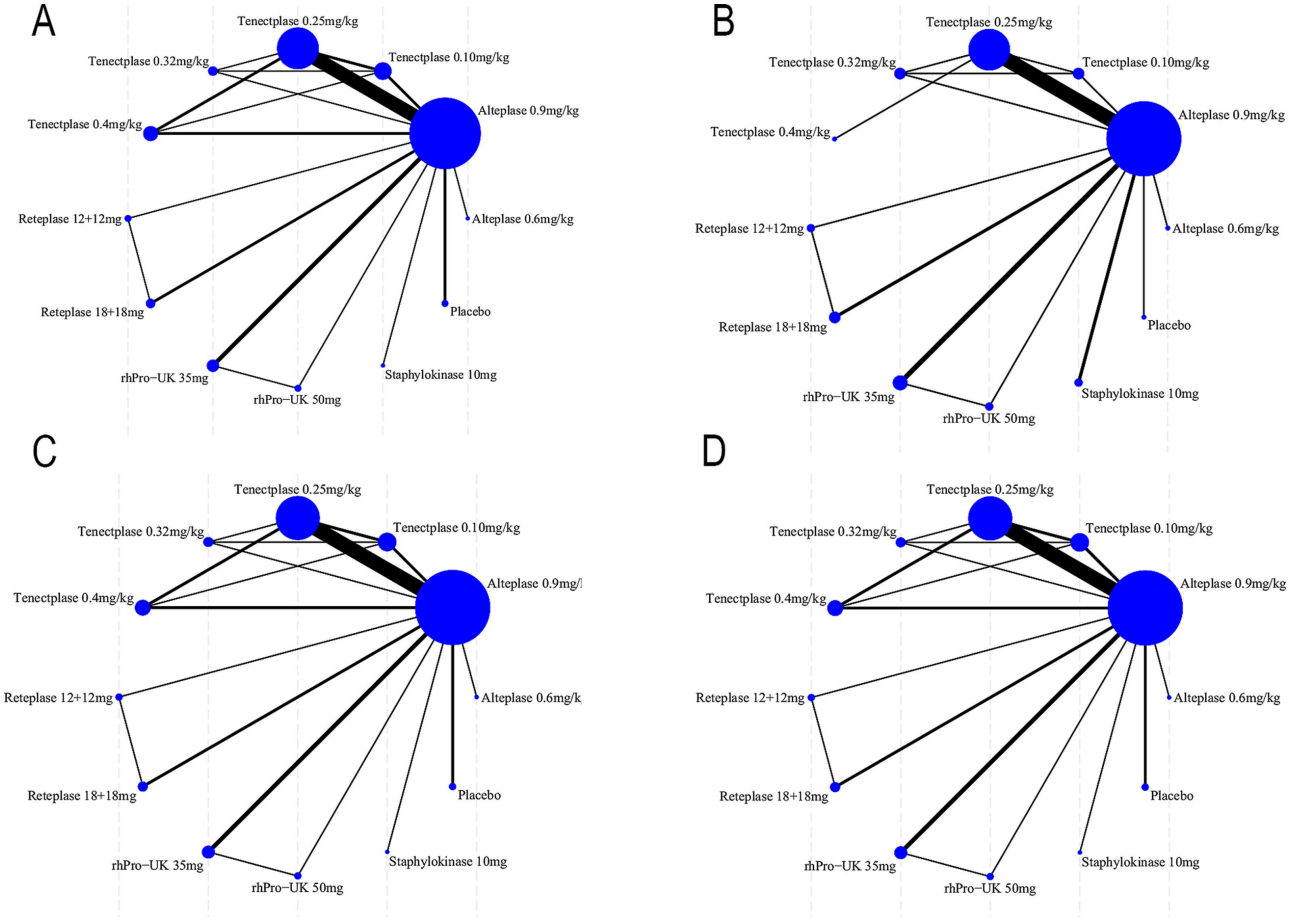
Evidence network diagram: **(A)** excellent functional outcome; **(B)** good functional outcome; **(C)** 90-day all-cause mortality events; **(D)** symptomatic intracranial hemorrhage events.

Figure 2 depicts the treatment network for excellent functional outcome, good functional outcome, mortality at 90 days, and sICH. Global consistency testing indicated no evidence of network inconsistency (mRS 0–1: *p* = 0.99; mRS 0–2: *p* = 0.66; mortality: *p* = 0.30; sICH: *p* = 0.33). Between-study heterogeneity was low for all outcomes (mRS 0–1: I^2^ = 29.3%, *p* = 0.06; mRS 0–2: I^2^ = 24.0%, *p* = 0.13; mortality: I^2^ = 9.6%, *p* = 0.31; sICH: I^2^ = 23.9%, *p* = 0.10). Given the absence of inconsistency and the consistently low heterogeneity, a fixed-effect consistency model was applied for all NMA.

### 3.2 Assessment of risk of bias

**Figure 3:**
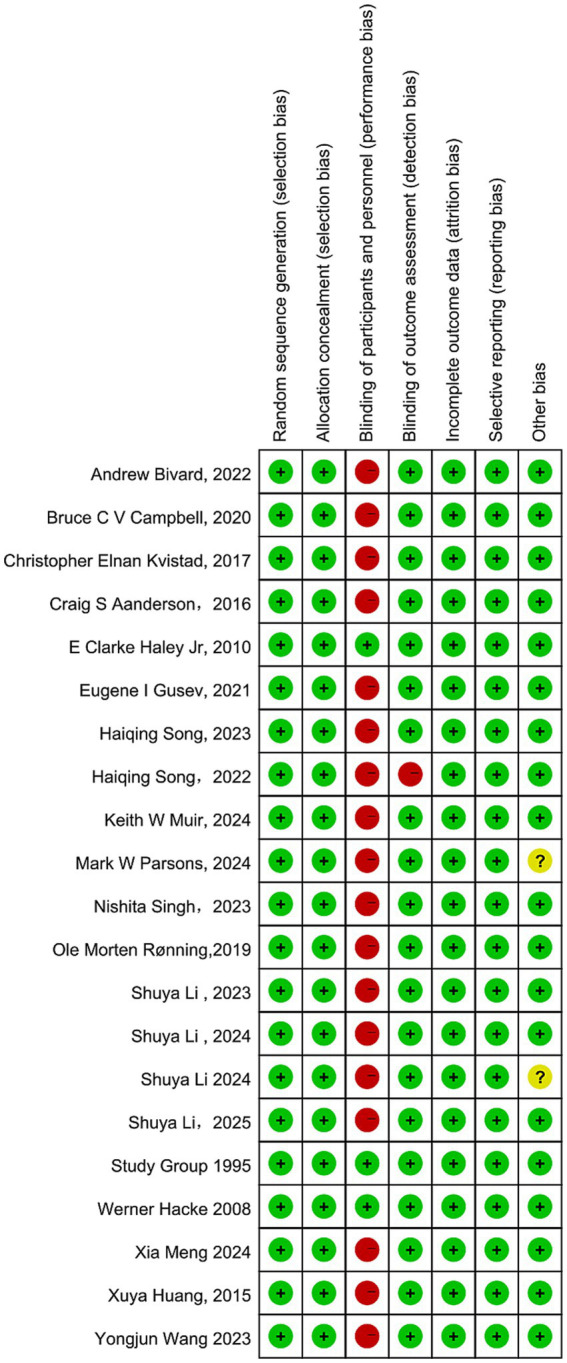
Cochrane risk of bias tool.

Figure 3 summarizes the risk of bias across the included studies. Most RCTs exhibited a high risk of performance bias, primarily due to the open-label trial designs. Funnel plots for each outcome are presented in Supplementary Figure 1, and Egger’ s tests revealed no significant evidence of small-study effects (Supplementary Table 2), suggesting that publication bias had minimal influence on the overall findings. According to the CINeMA evaluation, most pairwise comparisons were judged to have low confidence in the evidence. According to the CINeMA evaluation, most pairwise comparisons were judged to have low confidence in the evidence (Supplementary Tables 3–6).

To assess the transitivity assumption, we compared the distribution of key clinical variables across treatment comparisons, including baseline age, course of disease, and sample size. No evidence of violation of transitivity was identified when comparing the primary outcome profiles across different intravenous thrombolytic regimens (Supplementary Figure 2). Owing to limited data or inadequate reporting of potential effect modifiers across studies, detecting a certain degree of intransitivity using the available data may not be feasible.

### 3.3 Synthesized findings

#### 3.3.1 Excellent (mRS 0–1) functional outcome

The NMA demonstrated that, for achieving mRS 0–1 in patients with AIS, reteplase 18 + 18 mg was significantly more effective than several comparators: alteplase 0.6 mg/kg (OR = 1.74; 95% CI, 1.33–2.28), alteplase 0.9 mg/kg (OR = 1.60; 95% CI, 1.27–2.02), tenecteplase 0.25 mg/kg (OR = 1.48; 95% CI, 1.15–1.90) and rhPro-UK 35 mg (OR = 1.42; 95% CI, 1.06–1.90). Additionally, non-immunogenic recombinant staphylokinase 10 mg showed a statistically significant advantage over alteplase 0.6 mg/kg (OR = 2.43; 95% CI, 1.52–3.87), alteplase 0.9 mg/kg (OR = 2.23; 95% CI, 1.43–3.48), tenecteplase 0.10 mg/kg (OR = 2.39; 95% CI, 1.22–4.70), tenecteplase 0.25 mg/kg (OR = 2.06; 95% CI, 1.31–3.25), tenecteplase 0.32 mg/kg (OR = 2.25; 95% CI, 1.05–4.83), tenecteplase 0.40 mg/kg (OR = 2.09; 95% CI, 1.19–3.65), reteplase 12 + 12 mg (OR = 2.38; 95% CI, 1.07–5.30) and rhPro-UK 35 mg (OR = 1.98; 95% CI, 1.23–3.20). Furthermore, placebo was significantly inferior to alteplase 0.9 mg/kg (OR = 0.72; 95% CI, 0.56–0.92), tenecteplase 0.25 mg/kg (OR = 0.67; 95% CI, 0.51–0.87), reteplase 18 + 18 mg (OR = 0.45; 95% CI, 0.32–0.63), rhPro-UK 35 mg (OR = 0.64; 95% CI, 0.47–0.87). All other comparisons yielded no statistically significant differences (Table 2).

**Table 2:** League diagram of excellent functional outcome at 90 days.

Alteplase 0.6 mg/kg	Alteplase 0.9 mg/kg	Tenecteplase 0.10 mg/kg	Tenecteplase 0.25 mg/kg	Tenecteplase 0.32 mg/kg	Tenecteplase 0.40 mg/kg	Reteplase 12 + 12 mg	Reteplase 18 + 18 mg	rhPro-UK 35 mg	rhPro-UK 50 mg	Staphylokinase 10 mg	Placebo
Alteplase 0.6 mg/kg	1.09 (0.95, 1.25)	1.01 (0.60, 1.71)	1.18 (1.00, 1.39)	1.08 (0.57, 2.04)	1.16 (0.81, 1.68)	1.02 (0.52, 2.01)	1.74 (1.33, 2.28)	1.23 (0.98, 1.53)	1.35 (0.60, 3.02)	2.43 (1.52, 3.87)	0.78 (0.59, 1.04)
0.92 (0.80, 1.06)	Alteplase 0.9 mg/kg	0.93 (0.56, 1.55)	1.08 (0.98, 1.19)	0.99 (0.53, 1.85)	1.07 (0.76, 1.50)	0.94 (0.48, 1.82)	1.60 (1.27, 2.02)	1.13 (0.94, 1.34)	1.24 (0.56, 2.74)	2.23 (1.43, 3.48)	0.72 (0.56, 0.92)
0.99 (0.58, 1.67)	1.07 (0.65, 1.78)	Tenecteplase 0.10 mg/kg	1.16 (0.70, 1.92)	1.07 (0.54, 2.10)	1.15 (0.64, 2.07)	1.01 (0.44, 2.32)	1.72 (0.98, 2.99)	1.21 (0.71, 2.07)	1.33 (0.52, 3.41)	2.39 (1.22, 4.70)	0.77 (0.44, 1.36)
0.85 (0.72, 1.00)	0.92 (0.84, 1.02)	0.86 (0.52, 1.43)	Tenecteplase 0.25 mg/kg	0.92 (0.49, 1.71)	0.99 (0.71, 1.38)	0.87 (0.44, 1.70)	1.48 (1.15, 1.90)	1.04 (0.85, 1.27)	1.15 (0.51, 2.55)	2.06 (1.31, 3.25)	0.67 (0.51, 0.87)
0.93 (0.49, 1.75)	1.01 (0.54, 1.87)	0.94 (0.48, 1.85)	1.09 (0.58, 2.03)	Tenecteplase 0.32 mg/kg	1.08 (0.53, 2.17)	0.94 (0.38, 2.35)	1.61 (0.83, 3.13)	1.13 (0.59, 2.16)	1.25 (0.45, 3.42)	2.25 (1.05, 4.83)	0.73 (0.37, 1.42)
0.86 (0.60, 1.24)	0.94 (0.67, 1.31)	0.87 (0.48, 1.57)	1.01 (0.72, 1.42)	0.93 (0.46, 1.87)	Tenecteplase 0.40 mg/kg	0.88 (0.42, 1.85)	1.50 (0.99, 2.26)	1.05 (0.72, 1.55)	1.16 (0.49, 2.75)	2.09 (1.19, 3.65)	0.67 (0.44, 1.03)
0.98 (0.50, 1.93)	1.07 (0.55, 2.07)	0.99 (0.43, 2.29)	1.15 (0.59, 2.26)	1.06 (0.43, 2.63)	1.14 (0.54, 2.40)	Reteplase 12 + 12 mg	1.70 (0.88, 3.30)	1.20 (0.60, 2.39)	1.32 (0.47, 3.72)	2.38 (1.07, 5.30)	0.77 (0.38, 1.56)
0.57 (0.44, 0.75)	0.63 (0.50, 0.79)	0.58 (0.33, 1.02)	0.68 (0.53, 0.87)	0.62 (0.32, 1.21)	0.67 (0.44, 1.01)	0.59 (0.30, 1.14)	Reteplase 18 + 18 mg	0.70 (0.53, 0.94)	0.77 (0.34, 1.77)	1.40 (0.84, 2.31)	0.45 (0.32, 0.63)
0.82 (0.65, 1.02)	0.89 (0.74, 1.06)	0.83 (0.48, 1.41)	0.96 (0.79, 1.17)	0.88 (0.46, 1.68)	0.95 (0.65, 1.39)	0.83 (0.42, 1.66)	1.42 (1.06, 1.90)	rhPro-UK 35 mg	1.10 (0.50, 2.43)	1.98 (1.23, 3.20)	0.64 (0.47, 0.87)
0.74 (0.33, 1.66)	0.81 (0.36, 1.79)	0.75 (0.29, 1.93)	0.87 (0.39, 1.94)	0.80 (0.29, 2.20)	0.86 (0.36, 2.05)	0.76 (0.27, 2.13)	1.29 (0.56, 2.95)	0.91 (0.41, 2.01)	rhPro-UK 50 mg	1.80 (0.72, 4.48)	0.58 (0.25, 1.34)
0.41 (0.26, 0.66)	0.45 (0.29, 0.70)	0.42 (0.21, 0.82)	0.48 (0.31, 0.76)	0.45 (0.21, 0.96)	0.48 (0.27, 0.84)	0.42 (0.19, 0.94)	0.72 (0.43, 1.18)	0.51 (0.31, 0.82)	0.56 (0.22, 1.38)	Staphylokinase 10 mg	0.32 (0.19, 0.54)
1.28 (0.96, 1.69)	1.39 (1.09, 1.77)	1.29 (0.74, 2.27)	1.50 (1.15, 1.95)	1.38 (0.71, 2.69)	1.48 (0.98, 2.26)	1.30 (0.64, 2.65)	2.22 (1.58, 3.11)	1.56 (1.16, 2.12)	1.72 (0.75, 3.95)	3.10 (1.86, 5.15)	Placebo

rhPro-UK, recombinant human prourokinase; Staphylokinase, non-immunogenic recombinant staphylokinase.

**Figure 4:**
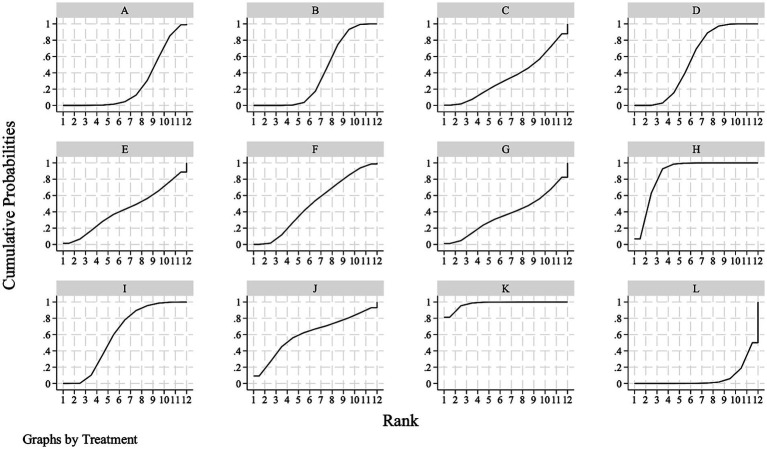
Surface under the cumulative ranking curve of excellent functional outcome at 90 days. **(A)** Alteplase 0.6 mg/kg; **(B)** alteplase 0.9 mg/kg; **(C)** tenecteplase 0.1 mg/kg; **(D)** tenecteplase 0.25 mg/kg; **(E)** tenecteplase 0.32 mg/kg; **(F)** tenecteplase 0.40 mg/kg; **(G)** reteplase 12 + 12 mg; **(H)** reteplase 18 + 18 mg; **(I)** recombinant human prourokinase 35 mg; **(J)** recombinant human prourokinase 50 mg; **(K)** non-immunogenic recombinant staphylokinase 10 mg; **(L)** placebo.

The SUCRAs for mRS 0–1 indicate that the top three ranking interventions included non-immunogenic recombinant staphylokinase 10 mg (97.8%), followed by reteplase 18 + 18 mg (87.5%) and rhPro-UK 50 mg (60.7%) (Figure 4; Table 3).

**Table 3:** Rank of different doses of thrombolytics on excellent functional outcome at 90 days.

Treatment	SUCRA	PrBest	MeanRank
Alteplase 0.6 mg/kg	26.9	0.0	9.0
Alteplase 0.9 mg/kg	39.7	0.0	7.6
Tenecteplase 0.1 mg/kg	34.3	0.2	8.2
Tenecteplase 0.25 mg/kg	56.0	0.0	5.8
Tenecteplase 0.32 mg/kg	42.2	1.1	7.4
Tenecteplase 0.40 mg/kg	50.7	0.1	6.4
Reteplase 12 + 12 mg	36.6	0.9	8.0
Reteplase 18 + 18 mg	87.5	7.2	2.4
rhPro-UK 35 mg	60.5	0.0	5.3
rhPro-UK 50 mg	60.7	9.2	5.3
Staphylokinase 10 mg	97.8	81.3	1.2
Placebo	7.1	0.0	11.2

SUCRA, surface under the cumulative ranking; PrBest, probability of being the best; rhPro-UK, Recombinant human prourokinase; Staphylokinase, Non-immunogenic recombinant staphylokinase.

#### 3.3.2 Good (mRS 0–2) functional outcome

The NMA showed that, for achieving mRS 0–2 in patients with AIS, reteplase 18 + 18 mg was significantly more effective than several comparators, including alteplase 0.6 mg/kg (OR = 1.44; 95% CI, 1.07–1.95), alteplase 0.9 mg/kg (OR = 1.41; 95% CI, 1.08–1.84), and non-immunogenic recombinant staphylokinase 10 mg (OR = 1.52; 95% CI, 1.06–2.18). No other comparisons demonstrated statistically significant differences (Table 4).

**Table 4:** League diagram of good functional outcome at 90 days.

Alteplase 0.6 mg	Alteplase 0.9 mg	Tenecteplase0.1 mg	Tenecteplase 0.25 mg	Tenecteplase 0.32 mg	Tenecteplase 0.40 mg	Reteplase 12 + 12 mg	Reteplase18 + 18 mg	rhPro-UK35 mg	rhPro-UK50 mg	Staphylokinase 10 mg	Placebo
Alteplase 0.6 mg	1.03 (0.89, 1.19)	0.83 (0.41, 1.67)	1.09 (0.91, 1.30)	0.83 (0.41, 1.67)	1.21 (0.74, 1.98)	0.76 (0.36, 1.60)	1.44 (1.07, 1.95)	1.05 (0.82, 1.34)	0.80 (0.35, 1.87)	0.95 (0.72, 1.26)	0.87 (0.54, 1.41)
0.97 (0.84, 1.12)	Alteplase 0.9 mg	0.81 (0.41, 1.60)	1.06 (0.96, 1.17)	0.81 (0.41, 1.60)	1.18 (0.74, 1.89)	0.74 (0.36, 1.54)	1.41 (1.08, 1.84)	1.02 (0.84, 1.24)	0.78 (0.34, 1.79)	0.92 (0.73, 1.18)	0.85 (0.54, 1.34)
1.20 (0.60, 2.42)	1.24 (0.62, 2.45)	Tenecteplase 0.1 mg	1.31 (0.66, 2.59)	1.00 (0.46, 2.16)	1.46 (0.64, 3.32)	0.92 (0.34, 2.49)	1.74 (0.84, 3.62)	1.26 (0.62, 2.57)	0.97 (0.33, 2.83)	1.14 (0.55, 2.36)	1.05 (0.46, 2.39)
0.92 (0.77, 1.10)	0.94 (0.85, 1.05)	0.76 (0.39, 1.51)	Tenecteplase 0.25 mg	0.76 (0.39, 1.51)	1.12 (0.71, 1.76)	0.70 (0.34, 1.46)	1.33 (1.00, 1.77)	0.97 (0.77, 1.21)	0.74 (0.32, 1.71)	0.87 (0.67, 1.13)	0.80 (0.51, 1.28)
1.20 (0.60, 2.42)	1.24 (0.62, 2.45)	1.00 (0.46, 2.16)	1.31 (0.66, 2.59)	Tenecteplase 0.32 mg	1.46 (0.64, 3.32)	0.92 (0.34, 2.49)	1.74 (0.84, 3.62)	1.26 (0.62, 2.57)	0.97 (0.33, 2.83)	1.14 (0.55, 2.36)	1.05 (0.46, 2.39)
0.82 (0.51, 1.35)	0.85 (0.53, 1.35)	0.69 (0.30, 1.56)	0.90 (0.57, 1.42)	0.69 (0.30, 1.56)	Tenecteplase 0.40 mg	0.63 (0.26, 1.50)	1.19 (0.70, 2.04)	0.87 (0.52, 1.44)	0.66 (0.26, 1.72)	0.78 (0.46, 1.33)	0.72 (0.38, 1.39)
1.31 (0.62, 2.76)	1.35 (0.65, 2.79)	1.09 (0.40, 2.96)	1.43 (0.68, 2.98)	1.09 (0.40, 2.96)	1.59 (0.67, 3.78)	Reteplase 12 + 12 mg	1.90 (0.92, 3.90)	1.38 (0.65, 2.93)	1.06 (0.35, 3.18)	1.25 (0.58, 2.68)	1.15 (0.49, 2.71)
0.69 (0.51, 0.94)	0.71 (0.54, 0.93)	0.58 (0.28, 1.20)	0.75 (0.57, 1.00)	0.58 (0.28, 1.20)	0.84 (0.49, 1.44)	0.53 (0.26, 1.08)	Reteplase 18 + 18 mg	0.73 (0.52, 1.01)	0.56 (0.23, 1.33)	0.66 (0.46, 0.94)	0.61 (0.36, 1.02)
0.95 (0.75, 1.22)	0.98 (0.80, 1.19)	0.79 (0.39, 1.61)	1.04 (0.83, 1.29)	0.79 (0.39, 1.61)	1.16 (0.69, 1.92)	0.73 (0.34, 1.54)	1.38 (0.99, 1.92)	rhPro-UK 35 mg	0.77 (0.33, 1.76)	0.90 (0.66, 1.24)	0.83 (0.51, 1.37)
1.24 (0.54, 2.88)	1.28 (0.56, 2.93)	1.03 (0.35, 3.03)	1.35 (0.59, 3.12)	1.03 (0.35, 3.03)	1.51 (0.58, 3.91)	0.95 (0.31, 2.86)	1.80 (0.75, 4.29)	1.30 (0.57, 2.99)	rhPro-UK 50 mg	1.18 (0.50, 2.80)	1.09 (0.42, 2.80)
1.05 (0.80, 1.39)	1.08 (0.85, 1.38)	0.88 (0.42, 1.81)	1.14 (0.88, 1.49)	0.88 (0.42, 1.81)	1.28 (0.75, 2.16)	0.80 (0.37, 1.73)	1.52 (1.06, 2.18)	1.11 (0.81, 1.51)	0.85 (0.36, 2.01)	Staphylokinase 10 mg	0.92 (0.55, 1.54)
1.14 (0.71, 1.84)	1.17 (0.75, 1.85)	0.95 (0.42, 2.16)	1.24 (0.78, 1.98)	0.95 (0.42, 2.16)	1.39 (0.72, 2.66)	0.87 (0.37, 2.06)	1.65 (0.98, 2.80)	1.20 (0.73, 1.97)	0.92 (0.36, 2.37)	1.09 (0.65, 1.81)	Placebo

rhPro-UK, Recombinant human prourokinase; Staphylokinase, Non-immunogenic recombinant staphylokinase.

**Figure 5:**
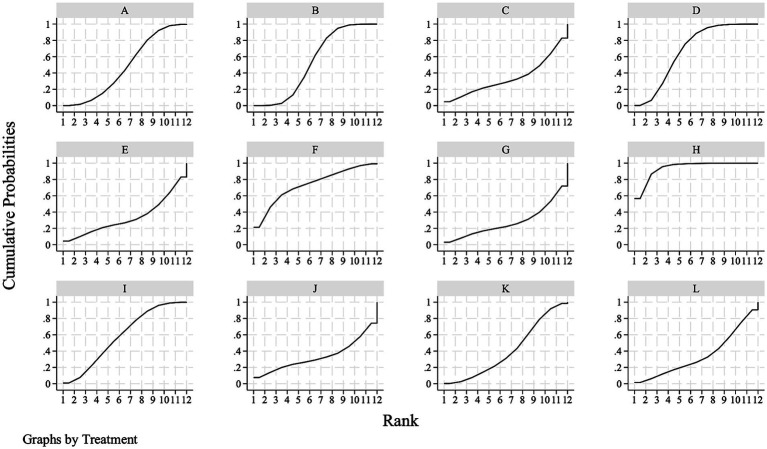
Surface under the cumulative ranking curve of good functional outcome at 90 days. **(A)** Alteplase 0.6 mg/kg; **(B)** alteplase 0.9 mg/kg; **(C)** tenecteplase 0.1 mg/kg; **(D)** tenecteplase 0.25 mg/kg; **(E)** tenecteplase 0.32 mg/kg; **(F)** tenecteplase 0.40 mg/kg; **(G)** reteplase 12 + 12 mg; **(H)** reteplase 18 + 18 mg; **(I)** recombinant human prourokinase 35 mg; **(J)** recombinant human prourokinase 50 mg; **(K)** non-immunogenic recombinant staphylokinase 10 mg; **(L)** placebo.

The SUCRAs for mRS 0–2 indicate that the top three ranking interventions included reteplase 18 + 18 mg (94.2%), followed by tenecteplase 0.40 mg/kg (74.4%) and tenecteplase 0.25 mg/kg (67.9%) (Figure 5; Table 5).

**Table 5:** Rank of different doses of thrombolytics on good functional outcome at 90 days.

Treatment	SUCRA	PrBest	MeanRank
Alteplase 0.6 mg	48.1	0.1	6.7
Alteplase 0.9 mg	53.5	0.0	6.1
Tenecteplase 0.1 mg	34.4	4.7	8.2
Tenecteplase 0.25 mg	67.9	0.2	4.5
Tenecteplase 0.32 mg	33.4	4.1	8.3
Tenecteplase 0.40 mg	74.4	22.6	3.8
Reteplase 12 + 12 mg	27.7	2.7	9.0
Reteplase 18 + 18 mg	94.2	56.7	1.6
rhPro-UK 35 mg	58.5	0.6	5.6
rhPro-UK 50 mg	33.1	6.8	8.4
Staphylokinase 10 mg	40.7	0.3	7.5
Placebo	34.3	1.3	8.2

SUCRA, surface under the cumulative ranking; PrBest, probability of being the best; rhPro-UK, Recombinant human prourokinase; Staphylokinase, Non-immunogenic recombinant staphylokinase.

#### 3.3.3 All-cause mortality at 90 days

There were no significant differences among the included treatments for all-cause mortality (Supplementary Table 7). The SUCRAs for all-cause mortality indicate that the top three ranking interventions included non-immunogenic recombinant staphylokinase 10 mg (23.3%), followed by alteplase 0.6 mg/kg (28.1%) and tenecteplase 0.1 mg/kg (30.1%) (Supplementary Figure 3; Supplementary Table 8).

#### 3.3.4 Symptomatic intracranial hemorrhage events

sICH events were evaluated in 21 studies involving 5 intravenous thrombolytics and 11 dosages, in addition to placebo. The NMA demonstrated that, non-immunogenic recombinant staphylokinase 10 mg significantly reduced the risk of sICH events: tenecteplase 0.25 mg/kg (OR = 0.31; 95% CI, 0.11–0.94), tenecteplase 0.40 mg/kg (OR = 0.18; 95% CI, 0.05–0.70) and reteplase 12 + 12 mg (OR = 0.11; 95% CI, 0.01–0.97). Additionally, placebo also significantly decreased risk of sICH events compared to these several comparators: alteplase 0.9 mg/kg (OR = 0.14; 95% CI, 0.04–0.46), tenecteplase 0.10 mg/kg (OR = 0.09; 95% CI, 0.01–0.63), tenecteplase 0.25 mg/kg (OR = 0.12; 95% CI, 0.02–0.41), tenecteplase 0.32 mg/kg (OR = 0.10; 95% CI, 0.01–0.85), tenecteplase 0.40 mg/kg (OR = 0.07; 95% CI, 0.02–0.30), reteplase 12 + 12 mg (OR = 0.04; 95% CI, 0.00–0.39) and reteplase 18 + 18 mg (OR = 0.12; 95% CI, 0.03–0.51). All other comparisons did not show statistically significant differences (Supplementary Table 9).

Based on the SUCRAs, placebo was associated with the lowest risk of sICH (2.2%), followed by non-immunogenic recombinant staphylokinase 10 mg (17.3%) and alteplase 0.6 mg/kg (22.3%) (Supplementary Figure 4; Supplementary Table 10).

### 3.4 Sensitivity analyses

Sensitivity analyses using random-effects models confirmed broad consistency with the primary analyses for excellent and good functional outcomes, all-cause mortality, and sICH (Supplementary Tables 11–14). Minor fluctuations in SUCRA values were noted for rhPro-UK 35 mg and rhPro-UK 50 mg regarding mRS 0–1 (Supplementary Figure 5; Supplementary Table 15), and for several regimens regarding mRS 0–2 (Supplementary Figure 6; Supplementary Table 16). Changes were also observed for alteplase 0.9 mg, tenecteplase 0.25 mg, and reteplase 12 + 12 mg in mortality outcomes (Supplementary Figure 7; Supplementary Table 17). No variations in SUCRA values were observed for sICH (Supplementary Figure 8; Supplementary Table 18).

### 3.5 Subgroup analysis

Subgroup analyses of patients achieving excellent and good functional outcomes indicated that reteplase was associated with a potential efficacy advantage over alteplase at 0.9 mg/kg (Supplementary Figures 9–10). However, no statistically significant differences between reteplase and alteplase 0.9 mg/kg were observed with respect to safety outcomes (Supplementary Figures 11–12). Likewise, rhPro-UK and tenecteplase demonstrated comparable efficacy and safety profiles to alteplase 0.9 mg/kg, with no significant differences detected across either efficacy or safety endpoints (Supplementary Figure 9–12).

## 4 Discussion

Our NMA, encompassing 21 RCTs and more than 16,000 patients, provides a comprehensive comparative framework for evaluating intravenous thrombolytic strategies administered within 4.5 h of AIS onset. Furthermore, the included studies demonstrated consistent baseline profiles, providing support for the transitivity assumption and enhancing the validity of the comparative estimates. A key finding is the identification of reteplase (18 + 18 mg) and non-immunogenic recombinant staphylokinase (10 mg) as promising alternatives that may surpass standard alteplase in achieving excellent functional outcomes (mRS 0–1). Notably, non-immunogenic recombinant staphylokinase also demonstrated a favorable safety profile, showing a markedly lower risk of sICH relative to several tenecteplase and reteplase regimens. Furthermore, no significant differences in all-cause mortality were observed across treatment arms, reinforcing the overall safety of thrombolytic therapy within this therapeutic window.

According to our NMA, there were statistically significant advantages in excellent and good functional outcomes for reteplase at 18 + 18 mg compared with alteplase, which is consistent with findings from recent meta-analyses. Hu et al., integrating 16 RCTs, similarly identified reteplase 18 + 18 mg as the top-ranked therapy (SUCRA 94.7%) (42). Sun et al. likewise reported significantly improved outcomes with reteplase relative to alteplase (OR 1.55, 95% CI 1.23–1.95) (43). This efficacy signal is further supported by direct comparisons: Alkhiri et al. demonstrated through pairwise meta-analysis that reteplase 18 + 18 mg confers a significantly higher likelihood of achieving excellent functional outcomes (44). The concordance of findings across both network and pairwise approaches enhances the credibility of reteplase 18 + 18 mg as a potent and clinically viable alternative to alteplase.

In our NMA, tenecteplase 0.25 mg/kg showed no significant advantage over alteplase, which differs from the results reported in several pairwise meta-analyses. This discrepancy underscores the methodological distinctions between analytical approaches. Earlier meta-analyses, including that by Kobeissi et al., found no significant difference between tenecteplase and alteplase, specifically for the 0.25 mg/kg dose (45). However, more recent and larger analyses by Wang et al. and Sheraz et al. reported modest but statistically significant improvements in excellent functional outcome with tenecteplase 0.25 mg/kg (OR 1.15 and 1.14, respectively) (46, 47). These discrepancies likely arise because pairwise analyses evaluate individual comparisons in isolation, whereas NMA contextualizes each treatment within a competitive multicomparator framework. This interpretation is aligned with findings from Srisurapanont et al., in which tenecteplase 0.25 mg/kg ranked highest in efficacy (SUCRA 68.0) yet did not demonstrate statistically significant advantages over alteplase in direct comparisons (48). Similar patterns were reported by Waseem et al., where tenecteplase 0.25 mg/kg showed marginal improvement under frequentist analysis but was not dominant when evaluated within a broader intervention network (49). Collectively, these findings suggest that while tenecteplase 0.25 mg/kg remains an effective and safe alternative to alteplase, its relative ranking decreases when evaluated alongside a wider range of thrombolytic agents.

To our knowledge, this NMA constitutes the most comprehensive synthesis of thrombolytic therapies for AIS to date, integrating data from 21 RCTs and more than 16,000 participants. A notable contribution of this study is its inclusion of non-immunogenic recombinant staphylokinase-an agent not previously incorporated into broad comparative frameworks-thereby addressing a critical gap in earlier analyses with narrower drug coverage (42, 43). The favorable safety profile of this agent, particularly its reduced risk of sICH, highlights its therapeutic potential for patients at elevated bleeding risk. Methodologically, the application of the CINeMA framework strengthens the validity of our findings by systematically evaluating heterogeneity, indirectness, and imprecision, thus providing a more reliable foundation for clinical decision-making.

Several limitations should be acknowledged. First, according to our CINeMA ratings, confidence in the findings was low for most comparisons, mainly due to uncertainty from the absence of a prespecified analysis plan, imprecise treatment effect estimates, and inadequate reporting of randomization and allocation concealment. Second, the small sample sizes for certain drugs, particularly non-immunogenic recombinant staphylokinase and specific tenecteplase doses, with some treatment nodes supported by only single small trials, may raise the potential for small-study effects. Therefore, the findings for these agents warrant cautious interpretation. Third, the use of disparate scales (e.g., ECASS III, ECASS II, SITS-MOST, and investigator-assessed criteria) to measure sICH across the included RCTs may introduce a risk of measurement bias arising from heterogeneity in outcome definitions. Fourth, the open-label design employed in several trials, together with potential conflicts of interest, may introduce performance bias and detection bias, which could disproportionately influence assessments of the mRS, a subjective clinical outcome.

This NMA provides a contemporary hierarchical assessment of thrombolytic therapies for patients with AIS treated within 4.5 h of symptom onset, indicating that reteplase (18 + 18 mg) and non-immunogenic recombinant staphylokinase (10 mg) were associated with the highest probabilities of improved functional outcomes. Notably, the limited number of randomized trials evaluating non-immunogenic recombinant staphylokinase precludes definitive conclusions regarding its efficacy and safety profile, necessitating future large-scale, high-quality studies to further establish these outcomes.

## Data Availability

The original contributions presented in the study are included in the article/Supplementary material, further inquiries can be directed to the corresponding author.

## Supplementary material

SUPPLEMENTARY FIGURE 1 Funnel plot. **(A)** Excellent functional outcome; **(B)** good functional outcome; **(C)** 90-day all-cause mortality events; **(D)** symptomatic intracranial hemorrhage events.

SUPPLEMENTARY FIGURE 2 Transitivity assessments in terms of distributions. **(A)** Age; **(B)** course of disease; **(C)** sample size across the studies.

SUPPLEMENTARY FIGURE 3 Surface under the cumulative ranking curve of 90-day all-cause mortality. **(A)** Alteplase 0.6 mg/kg; **(B)** alteplase 0.9 mg/kg; **(C)** tenecteplase 0.1 mg/kg; **(D)** tenecteplase 0.25 mg/kg; **(E)** tenecteplase 0.32 mg/kg; **(F)** tenecteplase 0.40 mg/kg; **(G)** reteplase 12+12 mg; **(H)** reteplase 18+18 mg; **(I)** recombinant human prourokinase 35 mg; **(J)** recombinant human prourokinase 50 mg; **(K)** non-immunogenic recombinant staphylokinase 10 mg; **(L)** placebo.

SUPPLEMENTARY FIGURE 4 Surface under the cumulative ranking curve of symptomatic intracranial hemorrhage events. **(A)** Alteplase 0.6 mg/kg; **(B)** alteplase 0.9 mg/kg; **(C)** tenecteplase 0.1 mg/kg; **(D)** tenecteplase 0.25 mg/kg; **(E)** tenecteplase 0.32 mg/kg; **(F)** tenecteplase 0.40 mg/kg; **(G)** reteplase 12+12 mg; **(H)** reteplase 18+18 mg; **(I)** recombinant human prourokinase 35 mg; **(J)** recombinant human prourokinase 50 mg; **(K)** non-immunogenic recombinant staphylokinase 10 mg; **(L)** placebo.

SUPPLEMENTARY FIGURE 5 Network sensitive-analysis of surface under the cumulative ranking curve of excellent functional outcome at 90 days. **(A)** Alteplase 0.6 mg/kg; **(B)** alteplase 0.9 mg/kg; **(C)** tenecteplase 0.1 mg/kg; **(D)** tenecteplase 0.25 mg/kg; **(E)** tenecteplase 0.32 mg/kg; **(F)** tenecteplase 0.40 mg/kg; **(G)** reteplase 12+12 mg; **(H)** reteplase 18+18 mg; **(I)** recombinant human prourokinase 35 mg; **(J)** recombinant human prourokinase 50 mg; **(K)** non-immunogenic recombinant staphylokinase 10 mg; **(L)** placebo.

SUPPLEMENTARY FIGURE 6 Network sensitive-analysis of surface under the cumulative ranking curve of good functional outcome at 90 days. **(A)** Alteplase 0.6 mg/kg; **(B)** alteplase 0.9 mg/kg; **(C)** tenecteplase 0.1 mg/kg; **(D)** tenecteplase 0.25 mg/kg; **(E)** tenecteplase 0.32 mg/kg; **(F)** tenecteplase 0.40 mg/kg; **(G)** reteplase 12+12 mg; **(H)** reteplase 18+18 mg; **(I)** recombinant human prourokinase 35 mg; **(J)** recombinant human prourokinase 50 mg; **(K)** non-immunogenic recombinant staphylokinase 10 mg; **(L)** placebo.

SUPPLEMENTARY FIGURE 7. Network sensitive-analysis of surface under the cumulative ranking curve of all-cause mortality events at 90 days. **(A)** Alteplase 0.6 mg/kg; **(B)** alteplase 0.9 mg/kg; **(C)** tenecteplase 0.1 mg/kg; **(D)** tenecteplase 0.25 mg/kg; **(E)** tenecteplase 0.32 mg/kg; **(F)** tenecteplase 0.40 mg/kg; **(G)** reteplase 12+12 mg; **(H)** reteplase 18+18 mg; **(I)** recombinant human prourokinase 35 mg; **(J)** recombinant human prourokinase 50 mg; **(K)** non-immunogenic recombinant staphylokinase 10 mg; **(L)** placebo.

SUPPLEMENTARY FIGURE 8 Network sensitive-analysis of surface under the cumulative ranking curve of symptomatic intracranial hemorrhage events. **(A)** Alteplase 0.6 mg/kg; **(B)** alteplase 0.9 mg/kg; **(C)** tenecteplase 0.1 mg/kg; **(D)** tenecteplase 0.25 mg/kg; **(E)** tenecteplase 0.32 mg/kg; **(F)** tenecteplase 0.40 mg/kg; **(G)** reteplase 12+12 mg; **(H)** reteplase 18+18 mg; **(I)** recombinant human prourokinase 35 mg; **(J)** recombinant human prourokinase 50 mg; **(K)** non-immunogenic recombinant staphylokinase 10 mg; **(L)** placebo.

SUPPLEMENTARY FIGURE 9 Forest plot for the subgroup analysis of excellent functional outcome at 90 days.

SUPPLEMENTARY FIGURE 10 Forest plot for the subgroup analysis of good functional outcome at 90 days.

SUPPLEMENTARY FIGURE 11 Forest plot for the subgroup analysis of 90-day all-cause mortality events.

SUPPLEMENTARY FIGURE 12 Forest plot for the subgroup analysis of symptomatic intracranial hemorrhage events.
