# Supplementary figures and images for: Comparison of the efficacy and safety of different thrombolytic drugs in the treatment of acute ischemic stroke within 4.5 h: a systematic review and network meta-analysis
Source: Front Neurol. 2026 Mar 27;17:1775325. doi: 10.3389/fneur.2026.1775325 (PMC13065716; doi:10.3389/fneur.2026.1775325)

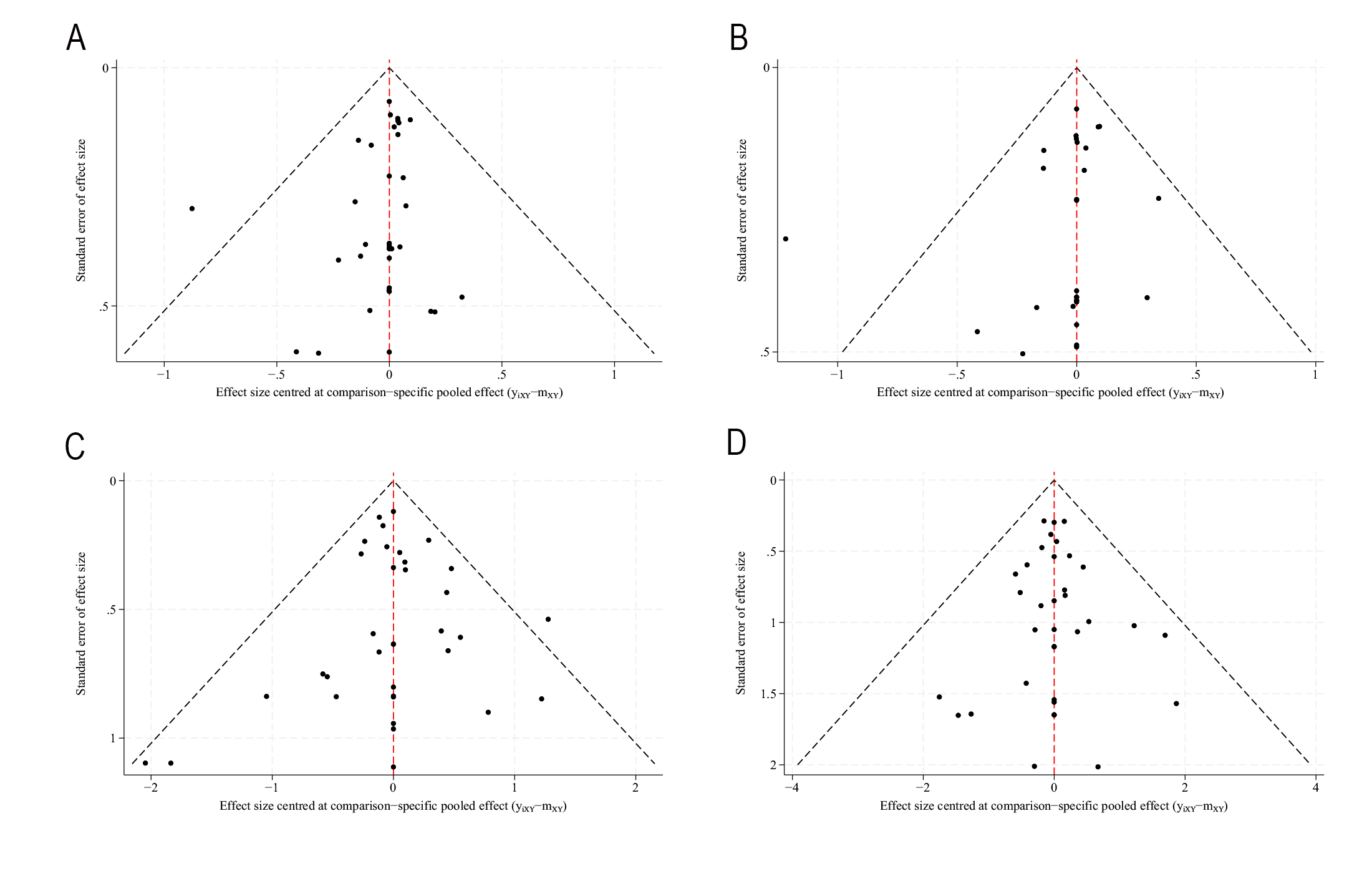

Supplement: SUPPLEMENTARY FIGURE 1 — Funnel plot. (A) Excellent functional outcome; (B) good functional outcome; (C) 90-day all-cause mortality events; (D) symptomatic intracranial hemorrhage events. [file Image_1.TIF]

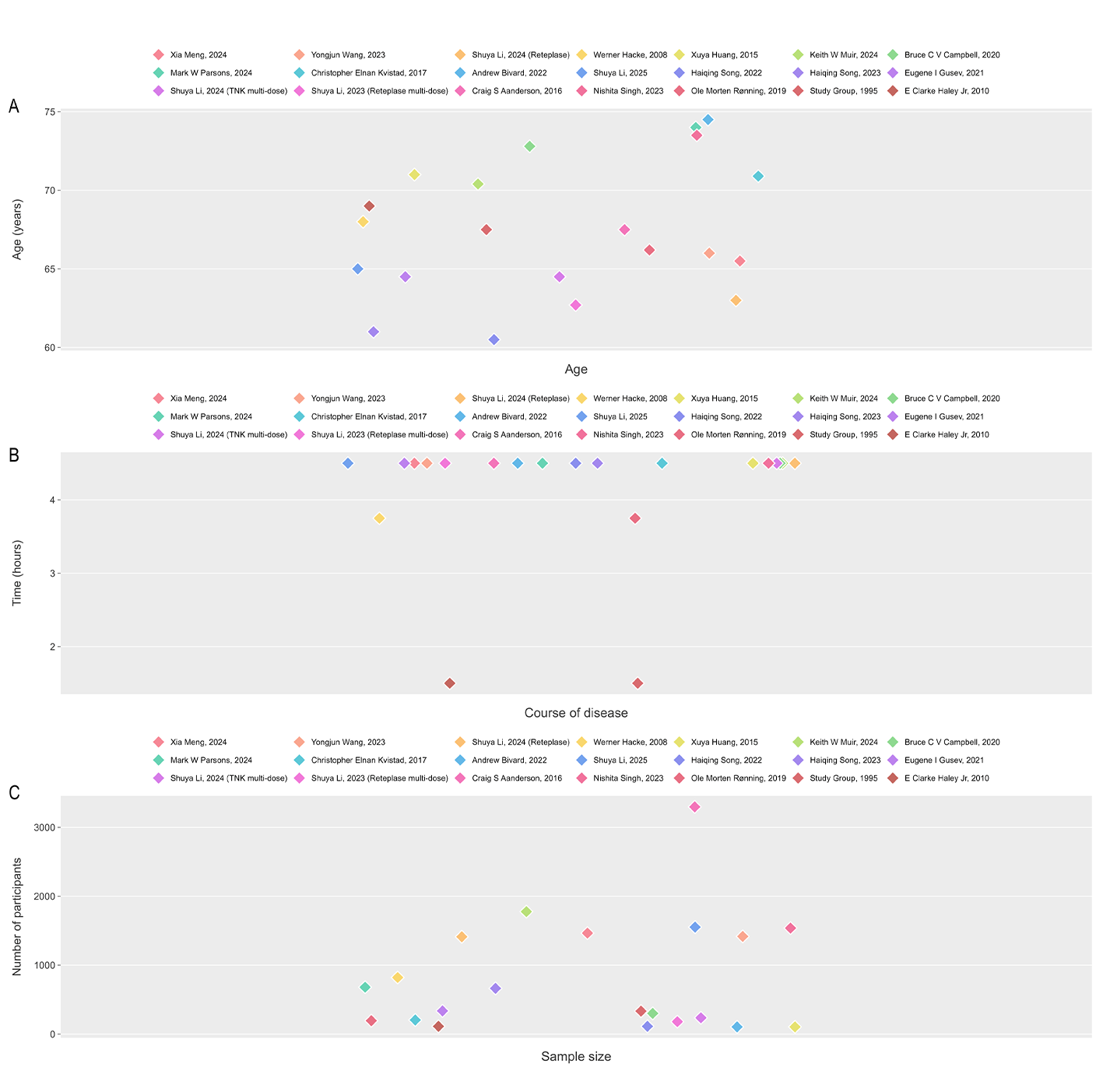

Supplement: SUPPLEMENTARY FIGURE 2 — Transitivity assessments in terms of distributions. (A) Age; (B) course of disease; (C) sample size across the studies. [file Image_2.TIFF]

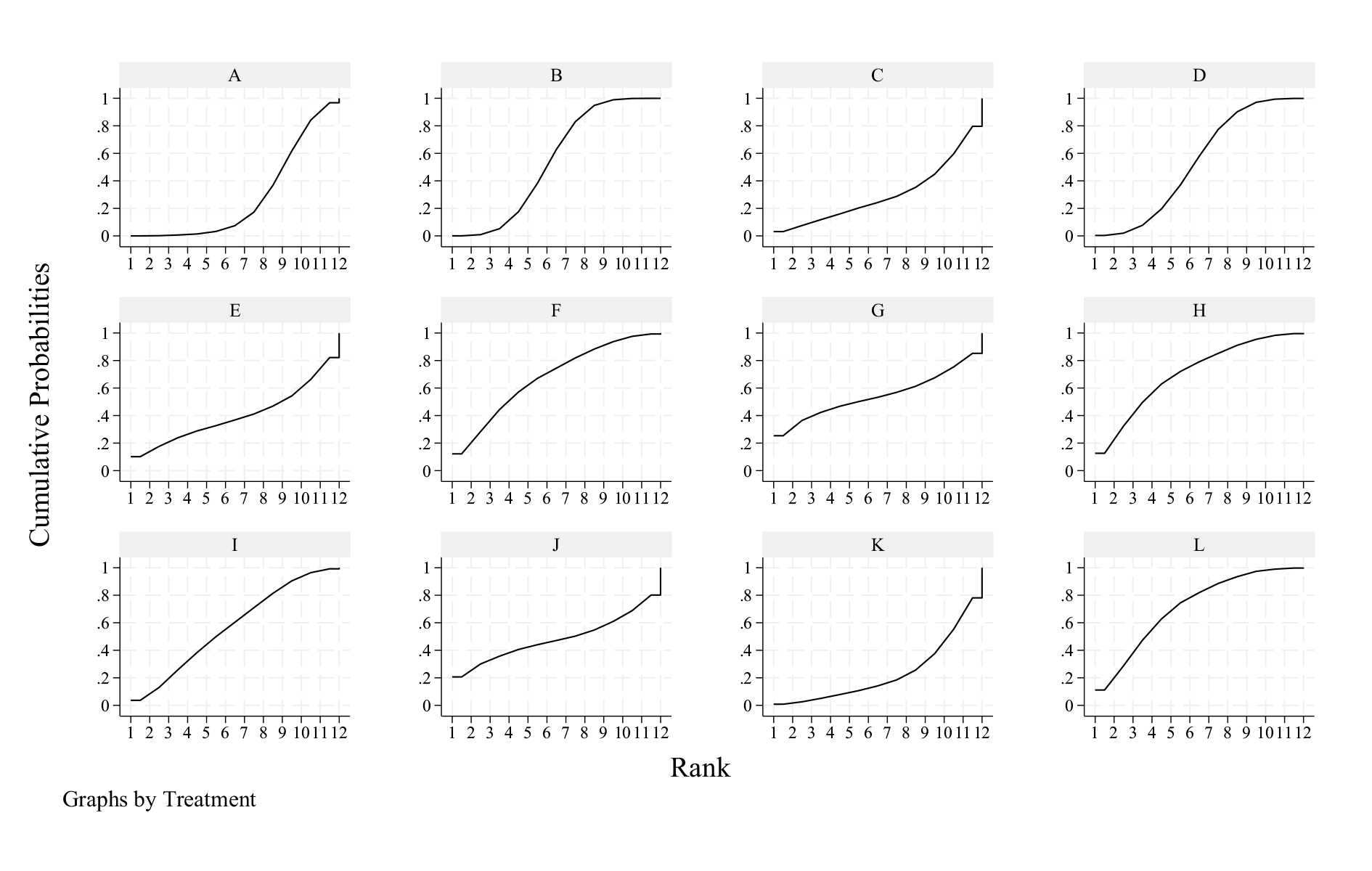

Supplement: SUPPLEMENTARY FIGURE 3 — Surface under the cumulative ranking curve of 90-day all-cause mortality. (A) Alteplase 0.6 mg/kg; (B) alteplase 0.9 mg/kg; (C) tenecteplase 0.1 mg/kg; (D) tenecteplase 0.25 mg/kg; (E) tenecteplase 0.32 mg/kg; (F) tenecteplase 0.40 mg/kg; (G) reteplase 12+12 mg; (H) reteplase 18+18 mg; (I) recombinant human prourokinase 35 mg; (J) recombinant human prourokinase 50 mg; (K) non-immunogenic recombinant staphylokinase 10 mg; (L) placebo. [file Image_3.TIF]

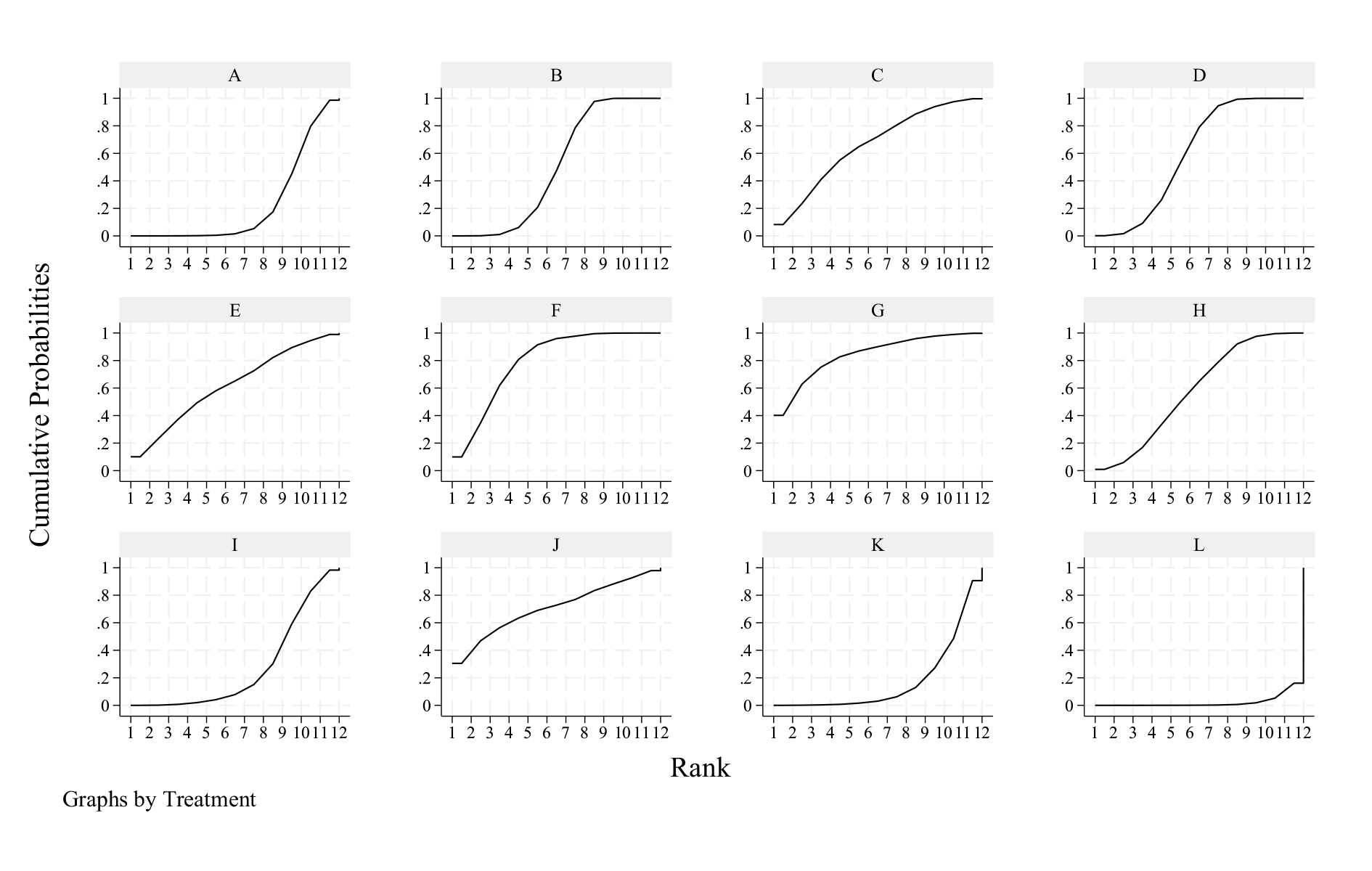

Supplement: SUPPLEMENTARY FIGURE 4 — Surface under the cumulative ranking curve of symptomatic intracranial hemorrhage events. (A) Alteplase 0.6 mg/kg; (B) alteplase 0.9 mg/kg; (C) tenecteplase 0.1 mg/kg; (D) tenecteplase 0.25 mg/kg; (E) tenecteplase 0.32 mg/kg; (F) tenecteplase 0.40 mg/kg; (G) reteplase 12+12 mg; (H) reteplase 18+18 mg; (I) recombinant human prourokinase 35 mg; (J) recombinant human prourokinase 50 mg; (K) non-immunogenic recombinant staphylokinase 10 mg; (L) placebo. [file Image_4.TIF]

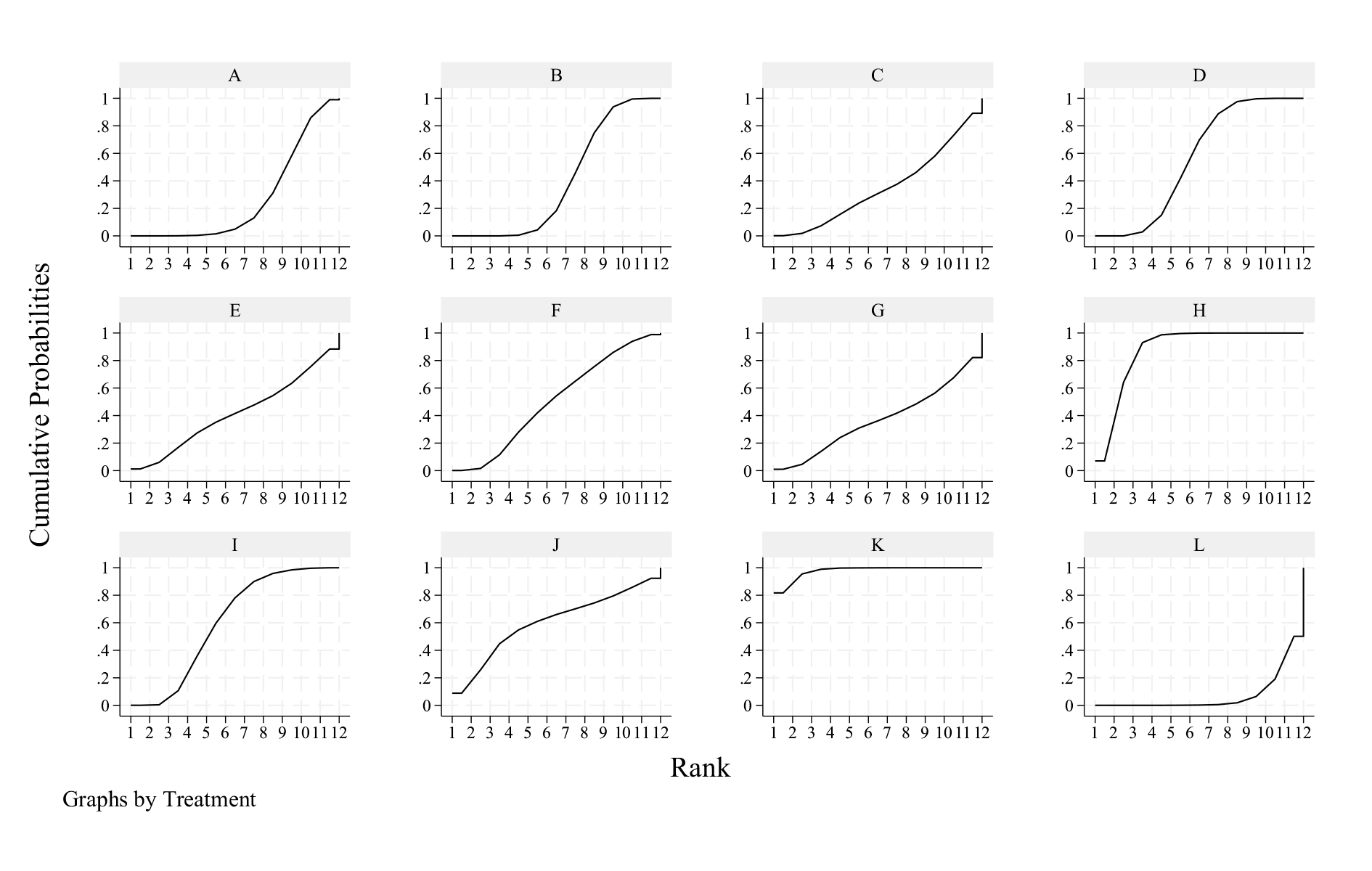

Supplement: SUPPLEMENTARY FIGURE 5 — Network sensitive-analysis of surface under the cumulative ranking curve of excellent functional outcome at 90 days. (A) Alteplase 0.6 mg/kg; (B) alteplase 0.9 mg/kg; (C) tenecteplase 0.1 mg/kg; (D) tenecteplase 0.25 mg/kg; (E) tenecteplase 0.32 mg/kg; (F) tenecteplase 0.40 mg/kg; (G) reteplase 12+12 mg; (H) reteplase 18+18 mg; (I) recombinant human prourokinase 35 mg; (J) recombinant human prourokinase 50 mg; (K) non-immunogenic recombinant staphylokinase 10 mg; (L) placebo. [file Image_5.TIF]

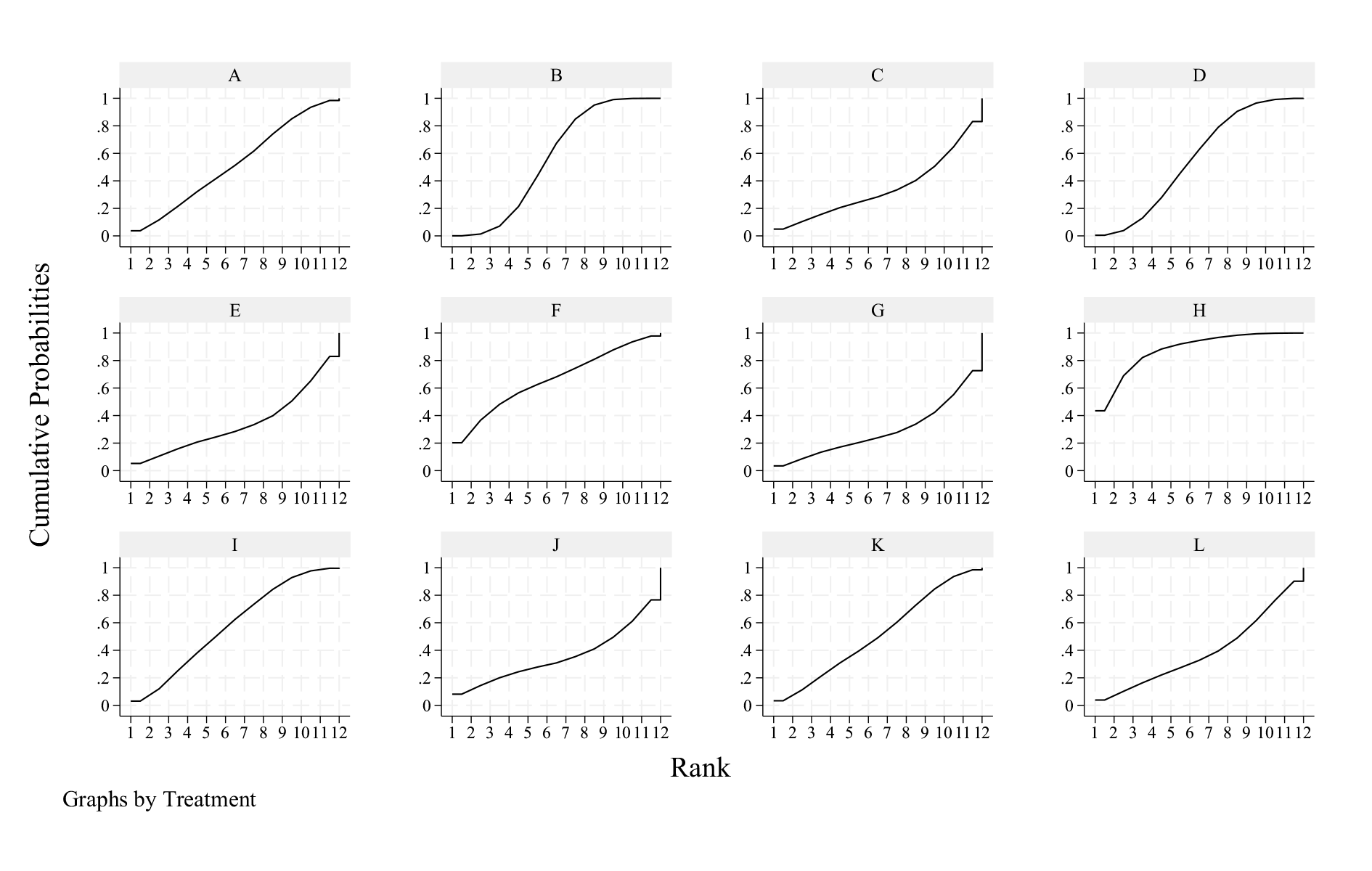

Supplement: SUPPLEMENTARY FIGURE 6 — Network sensitive-analysis of surface under the cumulative ranking curve of good functional outcome at 90 days. (A) Alteplase 0.6 mg/kg; (B) alteplase 0.9 mg/kg; (C) tenecteplase 0.1 mg/kg; (D) tenecteplase 0.25 mg/kg; (E) tenecteplase 0.32 mg/kg; (F) tenecteplase 0.40 mg/kg; (G) reteplase 12+12 mg; (H) reteplase 18+18 mg; (I) recombinant human prourokinase 35 mg; (J) recombinant human prourokinase 50 mg; (K) non-immunogenic recombinant staphylokinase 10 mg; (L) placebo. [file Image_6.TIF]

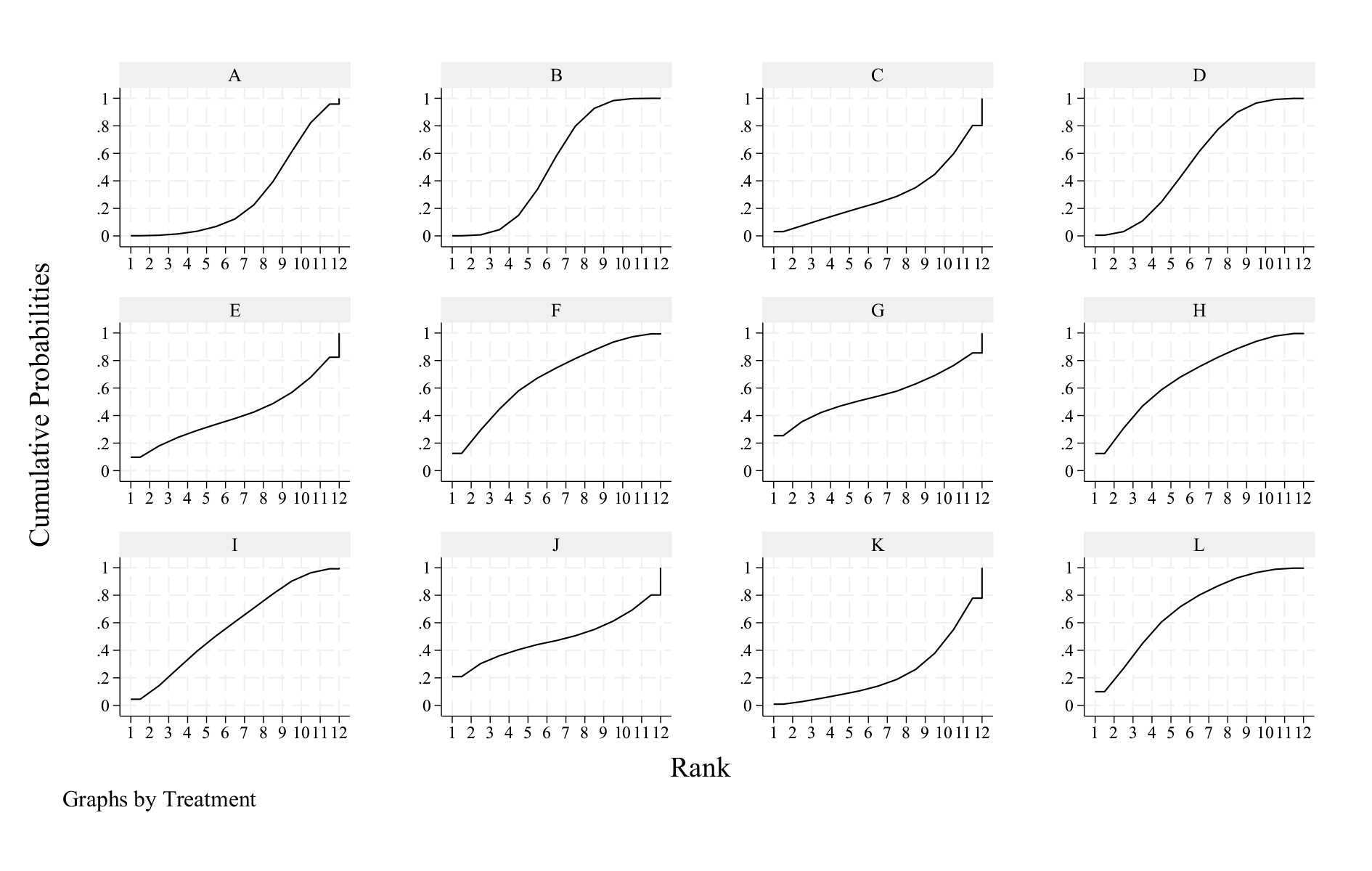

Supplement: SUPPLEMENTARY FIGURE 7. — Network sensitive-analysis of surface under the cumulative ranking curve of all-cause mortality events at 90 days. (A) Alteplase 0.6 mg/kg; (B) alteplase 0.9 mg/kg; (C) tenecteplase 0.1 mg/kg; (D) tenecteplase 0.25 mg/kg; (E) tenecteplase 0.32 mg/kg; (F) tenecteplase 0.40 mg/kg; (G) reteplase 12+12 mg; (H) reteplase 18+18 mg; (I) recombinant human prourokinase 35 mg; (J) recombinant human prourokinase 50 mg; (K) non-immunogenic recombinant staphylokinase 10 mg; (L) placebo. [file Image_7.TIF]

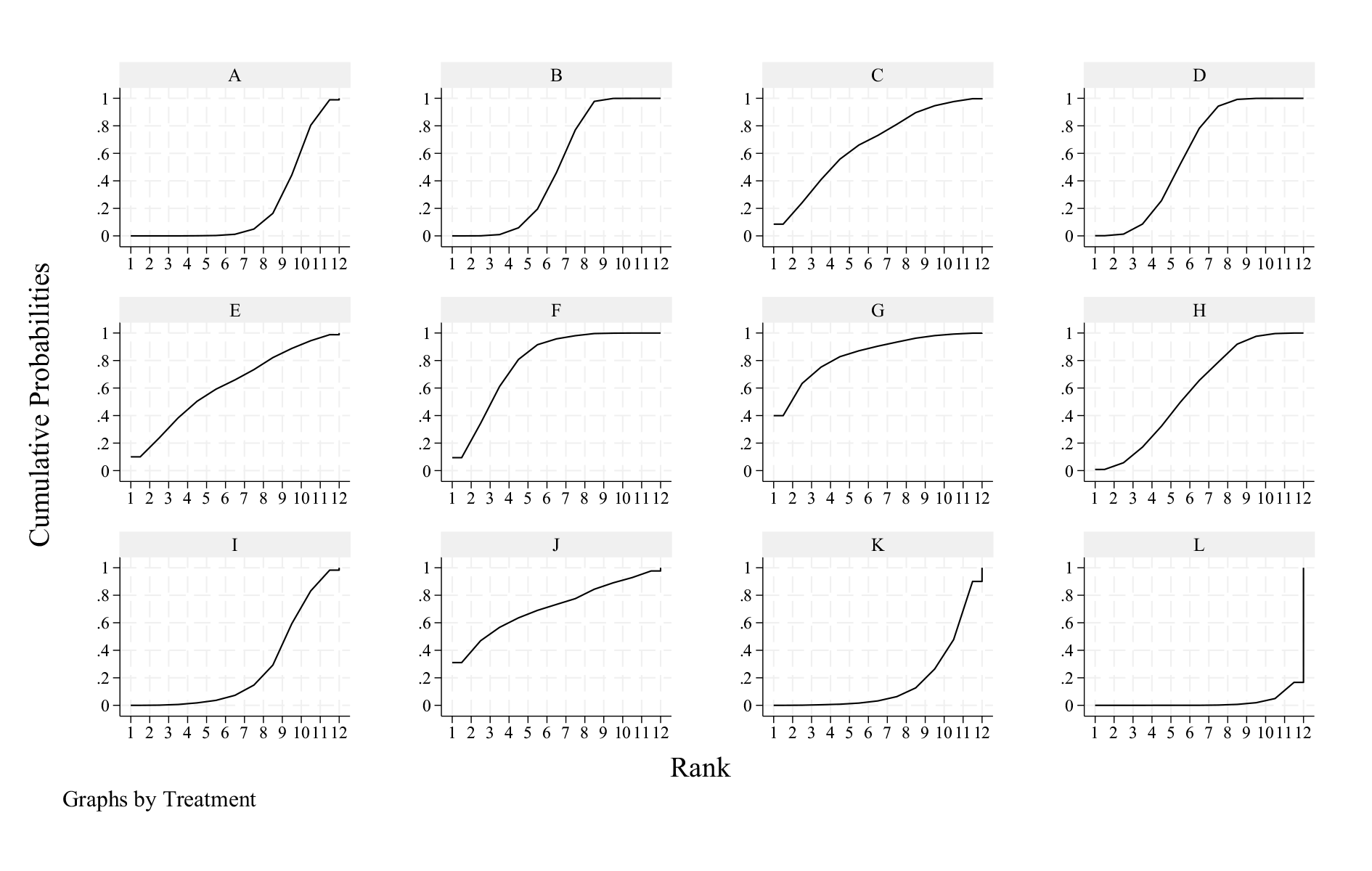

Supplement: SUPPLEMENTARY FIGURE 8 — Network sensitive-analysis of surface under the cumulative ranking curve of symptomatic intracranial hemorrhage events. (A) Alteplase 0.6 mg/kg; (B) alteplase 0.9 mg/kg; (C) tenecteplase 0.1 mg/kg; (D) tenecteplase 0.25 mg/kg; (E) tenecteplase 0.32 mg/kg; (F) tenecteplase 0.40 mg/kg; (G) reteplase 12+12 mg; (H) reteplase 18+18 mg; (I) recombinant human prourokinase 35 mg; (J) recombinant human prourokinase 50 mg; (K) non-immunogenic recombinant staphylokinase 10 mg; (L) placebo. [file Image_8.TIF]

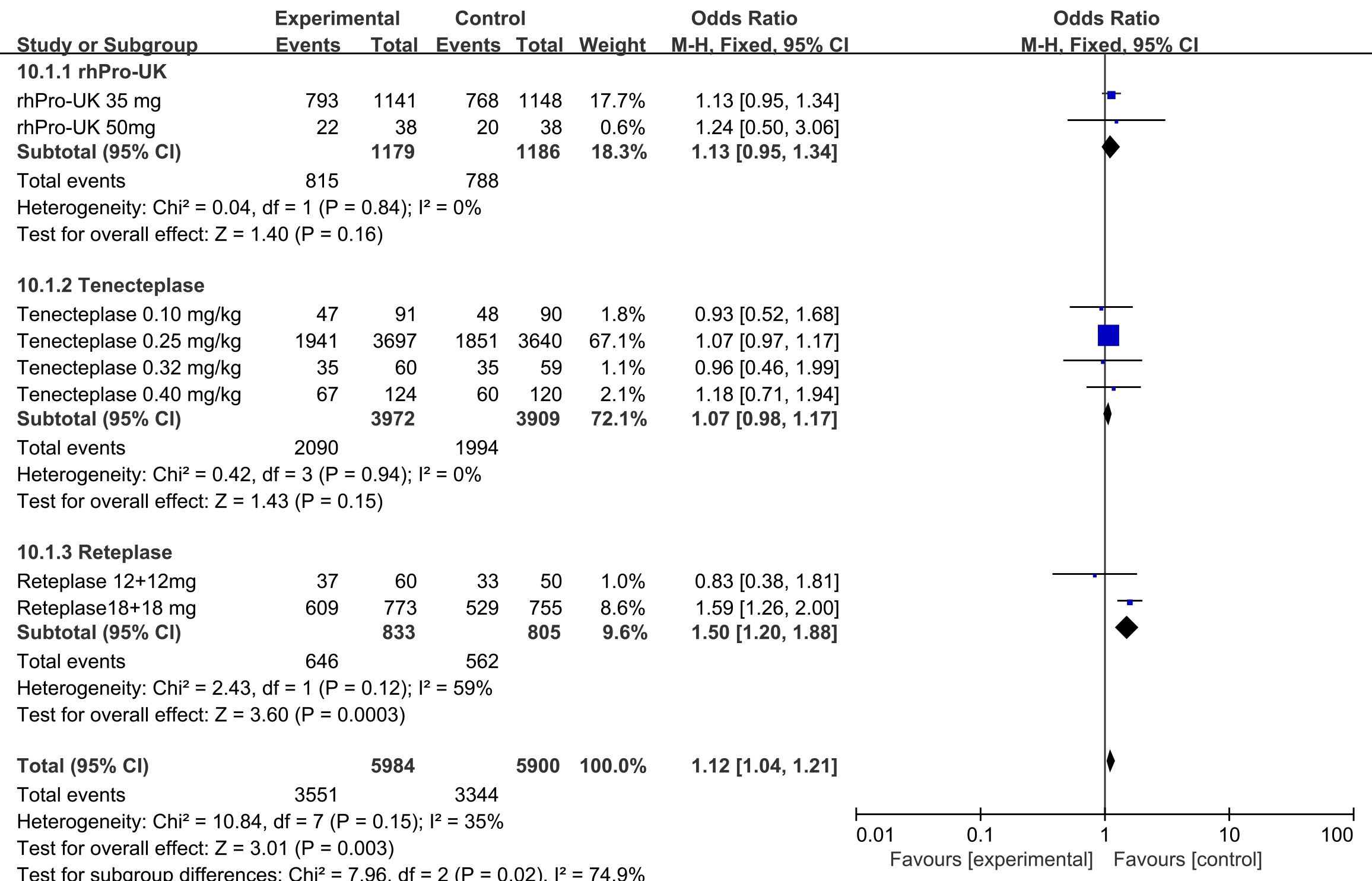

Supplement: SUPPLEMENTARY FIGURE 9 — Forest plot for the subgroup analysis of excellent functional outcome at 90 days. [file Image_9.TIF]

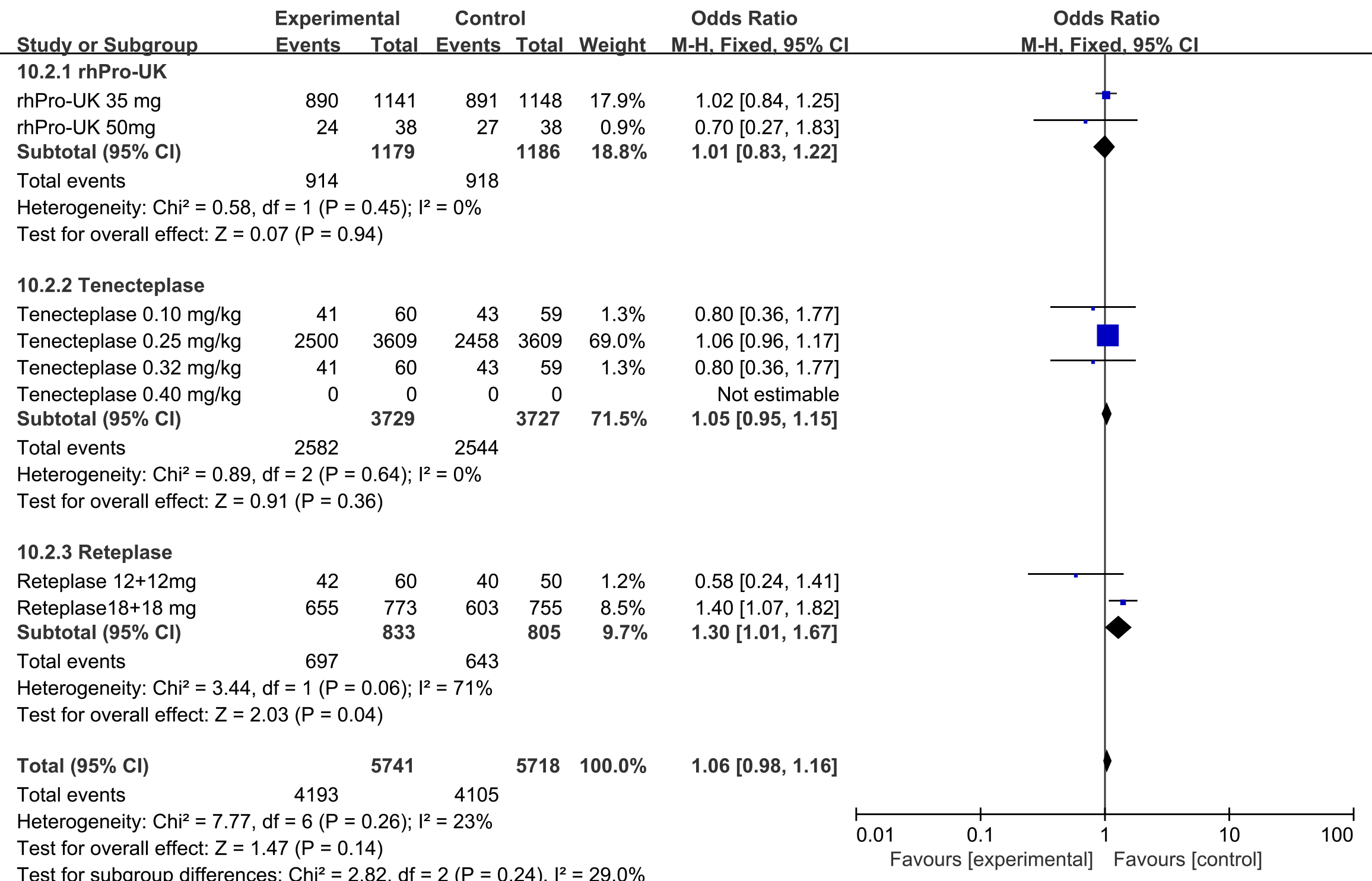

Supplement: SUPPLEMENTARY FIGURE 10 — Forest plot for the subgroup analysis of good functional outcome at 90 days. [file Image_10.TIF]

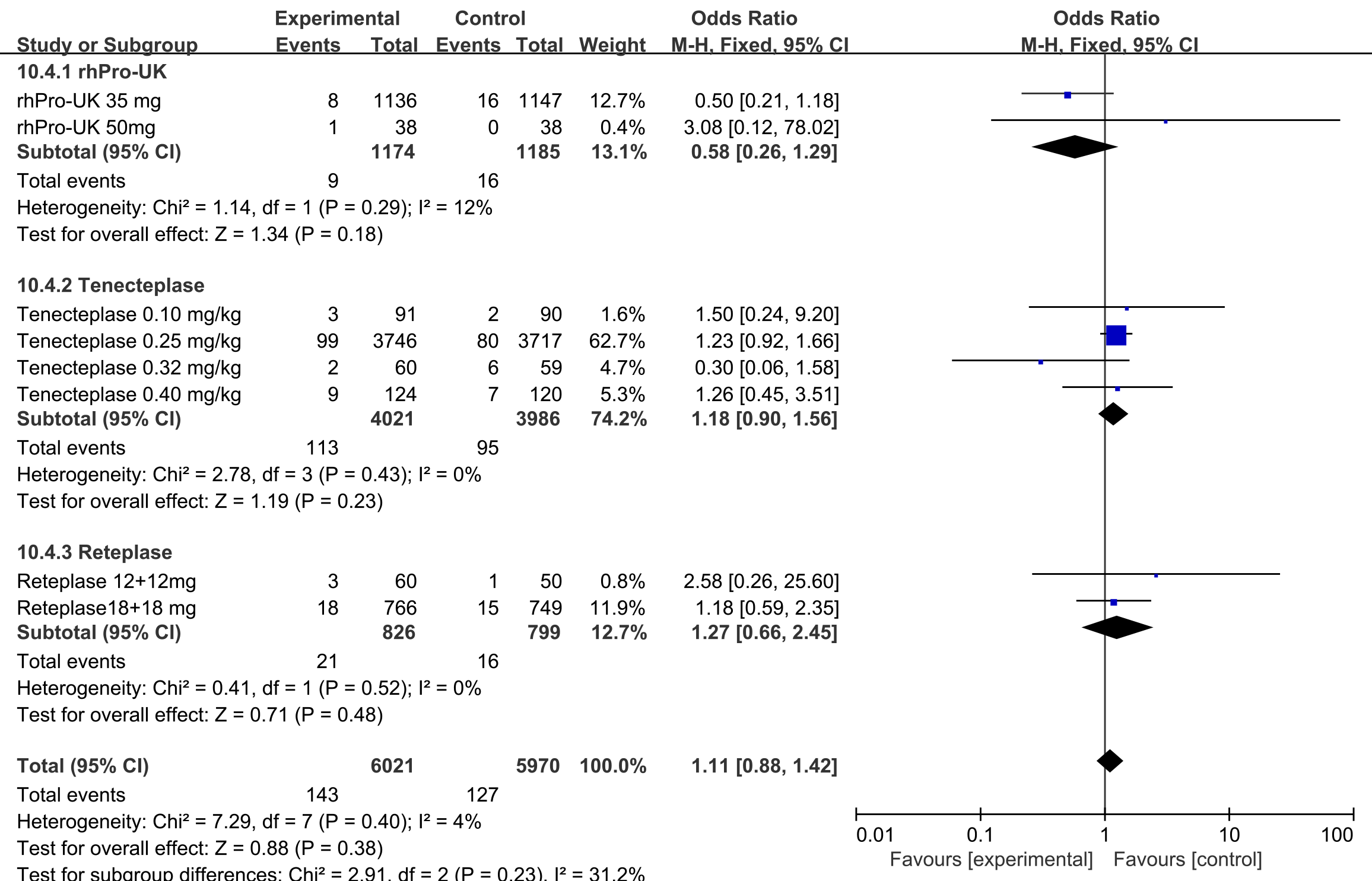

Supplement: SUPPLEMENTARY FIGURE 11 — Forest plot for the subgroup analysis of 90-day all-cause mortality events. [file Image_11.TIF]

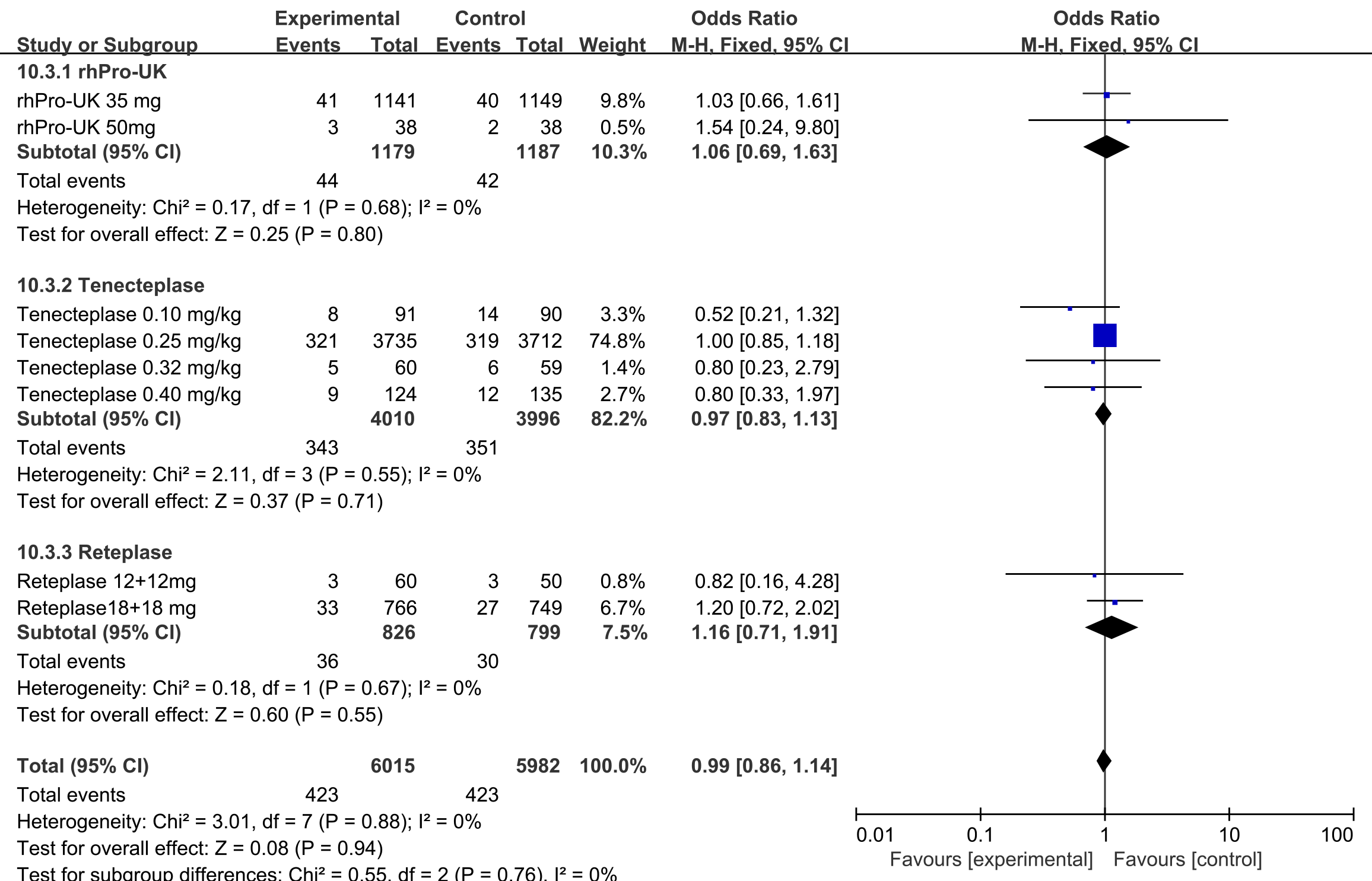

Supplement: SUPPLEMENTARY FIGURE 12 — Forest plot for the subgroup analysis of symptomatic intracranial hemorrhage events. [file Image_12.TIF]
