# Supplementary material for: Comparison of the efficacy and safety of different thrombolytic drugs in the treatment of acute ischemic stroke within 4.5 h: a systematic review and network meta-analysis
Source: Front Neurol. 2026 Mar 27;17:1775325. doi: 10.3389/fneur.2026.1775325 (PMC13065716; doi:10.3389/fneur.2026.1775325)
Supplement: Supplementary file 13 [file Table_1.DOCX]

**Supplementary Tables**

**Supplementary Table 1. The characteristics of the thrombolytic agents included in the study.**

| **Intervention** | **Dosage** | **Administration Method** |
| --- | --- | --- |
| Alteplase | 0.6 mg/kg | 0.6 mg/kg (maximum 60 mg), with 15% of the dose administered as an initial bolus, followed by 85% as a continuous infusion over 1 hour. |
|  | 0.9 mg/kg | 0.9 mg/kg (maximum 90 mg), with 10% of the dose administered as an initial bolus, followed by 90% as a continuous infusion over 1 hour. |
| Tenecteplase | 0.1 mg/kg | 0.1 mg/kg (maximum 10 mg) as a single bolus injection in 10 mL of normal saline, followed by 90 mL of normal saline infused over 1 hour. |
|  | 0.25 mg/kg | 0.25 mg/kg (maximum 25 mg) as a single bolus injection in 10 mL of normal saline, followed by 90 mL of normal saline infused over 1 hour. |
|  | 0.32 mg/kg | 0.32 mg/kg (maximum 32 mg) as a single bolus injection in 10 mL of normal saline, followed by 90 mL of normal saline infused over 1 hour. |
|  | 0.40 mg/kg | 0.40 mg/kg (maximum 40 mg) as a single bolus injection in 10 mL of normal saline, followed by 90 mL of normal saline infused over 1 hour. |
| Reteplase | 12+12 mg | 12 mg administered as an initial bolus, followed by a second 12 mg bolus after 30 minutes. |
|  | 18+18 mg | 18 mg administered as an initial bolus, followed by a second 18 mg bolus after 30 minutes. |
| rhPro-UK | 35 mg | 15 mg administered as an intravenous bolus over 3 minutes, with the remaining dose delivered as a continuous infusion over 30 minutes. |
|  | 50 mg | 20 mg administered as an intravenous bolus over 3 minutes, with the remaining dose delivered as a continuous infusion over 30 minutes. |
| Staphylokinase | 10 mg | A fixed 10 mg dose reconstituted in 10 mL of 0.9% sodium chloride, administered as a single intravenous bolus over 10 seconds. |
| Placebo | -- | -- |

Abbreviation: rhPro-UK, Recombinant human prourokinase; Staphylokinase, Non-immunogenic recombinant staphylokina

**Supplementary Table 2. Egger's test.**

| **Std_effect** | **Coefficient** | **Std. err.** | **t** | ***p*>\|t\|** | **95% CI** |
| --- | --- | --- | --- | --- | --- |
| Egger test of excellent functional outcome at 90 days |  |  |  |  |  |
| Slope | 0.1409897 | 0.0649747 | 2.17 | 0.037 | (0.0087976, 0.2731818) |
| Bias | -0.3002595 | 0.3484121 | -0.86 | 0.395 | (-1.009109, 0.4085903) |
| Test of H_0_: no small-study effects *P*=0.395 |  |  |  |  |  |
| Egger test of good functional outcome at 90 days |  |  |  |  |  |
| Slope | 0.1344036 | 0.0698256 | 1.92 | 0.065 | (-0.0091249, 0.2779321) |
| Bias | -0.6155058 | 0.3881745 | -1.59 | 0.125 | (-1.41341, 0.1823983) |
| Test of H_0_: no small-study effects *P*=0.125 |  |  |  |  |  |
| Egger test of all-cause mortality events at 90 days |  |  |  |  |  |
| Slope | 0.0760372 | 0.1020941 | 0.74 | 0.462 | (-0.1316748, 0.2837493) |
| Bias | -0.0725787 | 0.3036259 | -0.24 | 0.813 | (-0.6903104, 0.5451529) |
| Test of H_0_: no small-study effects *P*=0.813 |  |  |  |  |  |
| Egger test of symptomatic intracranial hemorrhage events |  |  |  |  |  |
| Slope | 0.2037694 | 0.2355102 | 0.87 | 0.393 | (-0.2753797, 0.6829186) |
| Bias | -0.1152649 | 0.3673987 | -0.31 | 0.756 | (-0.8627431, 0.6322133) |
| Test of H_0_: no small-study effects *P*=0.756 |  |  |  |  |  |

Abbreviation: CI, confidence intervals

**Supplementary Table 3. CINeMA on excellent functional outcome at 90 days.**

| **Comparison** | **Number of studies** | **Within-study bias** | **Reporting bias** | **Indirectness** | **Imprecision** | **Heterogeneity** | **Incoherence** | **Confidence rating** | **Reason(s) for downgrading** |
| --- | --- | --- | --- | --- | --- | --- | --- | --- | --- |
| Alteplase 0.6 mg/kg:Alteplase 0.9 mg/kg | 1 | No concerns | No concerns | No concerns | Major concerns | No concerns | No concerns | Low | Imprecision |
| Alteplase 0.9 mg/kg:Placebo | 2 | No concerns | No concerns | No concerns | No concerns | No concerns | No concerns | High | -- |
| Alteplase 0.9 mg/kg:rhPro-UK 50mg | 1 | No concerns | No concerns | No concerns | Major concerns | No concerns | No concerns | Low | Imprecision |
| Alteplase 0.9 mg/kg:Reteplase 12+12 mg | 1 | No concerns | No concerns | No concerns | Major concerns | No concerns | No concerns | Low | Imprecision |
| Alteplase 0.9 mg/kg:Reteplase 18+18 mg | 2 | No concerns | No concerns | No concerns | No concerns | No concerns | No concerns | High | -- |
| Alteplase 0.9 mg/kg:Staphylokinase 10mg | 1 | No concerns | No concerns | No concerns | No concerns | No concerns | No concerns | High | -- |
| Alteplase 0.9 mg/kg:Tenecteplase 0.1 mg/kg | 2 | No concerns | No concerns | No concerns | Major concerns | No concerns | No concerns | Low | Imprecision |
| Alteplase 0.9 mg/kg:Tenecteplase 0.25 mg/kg | 10 | No concerns | No concerns | No concerns | Major concerns | No concerns | No concerns | Low | Imprecision |
| Alteplase 0.9 mg/kg:Tenecteplase 0.32 mg/kg | 1 | No concerns | No concerns | No concerns | Major concerns | No concerns | No concerns | Low | Imprecision |
| Alteplase 0.9 mg/kg:Tenecteplase 0.40 mg/kg | 2 | No concerns | No concerns | No concerns | Major concerns | No concerns | No concerns | Low | Imprecision |
| Alteplase 0.9 mg/kg:rhPro-UK 35mg | 3 | No concerns | No concerns | No concerns | Major concerns | No concerns | No concerns | Low | Imprecision |
| rhPro-UK 50mg:rhPro-UK 35mg | 1 | No concerns | No concerns | No concerns | Major concerns | No concerns | No concerns | Low | Imprecision |
| Reteplase 12+12 mg:Reteplase 18+18 mg | 1 | No concerns | No concerns | No concerns | Major concerns | No concerns | No concerns | Low | Imprecision |
| Tenecteplase 0.1 mg/kg:Tenecteplase 0.25 mg/kg | 2 | No concerns | No concerns | No concerns | Major concerns | No concerns | No concerns | Low | Imprecision |
| Tenecteplase 0.1 mg/kg:Tenecteplase 0.32 mg/kg | 1 | No concerns | No concerns | No concerns | Major concerns | No concerns | No concerns | Low | Imprecision |
| Tenecteplase 0.1 mg/kg:Tenecteplase 0.40 mg/kg | 1 | No concerns | No concerns | No concerns | Major concerns | No concerns | No concerns | Low | Imprecision |
| Tenecteplase 0.25 mg/kg:Tenecteplase 0.32 mg/kg | 1 | No concerns | No concerns | No concerns | Major concerns | No concerns | No concerns | Low | Imprecision |
| Tenecteplase 0.25 mg/kg:Tenecteplase 0.40 mg/kg | 2 | No concerns | No concerns | No concerns | Major concerns | No concerns | No concerns | Low | Imprecision |
| Alteplase 0.6 mg/kg:Placebo | -- | No concerns | No concerns | No concerns | Major concerns | No concerns | No concerns | Low | Imprecision |
| Alteplase 0.6 mg/kg:rhPro-UK 50mg | -- | No concerns | No concerns | No concerns | Major concerns | No concerns | No concerns | Low | Imprecision |
| Alteplase 0.6 mg/kg:Reteplase 12+12 mg | -- | No concerns | No concerns | No concerns | Major concerns | No concerns | No concerns | Low | Imprecision |
| Alteplase 0.6 mg/kg:Reteplase 18+18 mg | -- | No concerns | No concerns | No concerns | No concerns | No concerns | No concerns | High | -- |
| Alteplase 0.6 mg/kg:Staphylokinase 10mg | -- | No concerns | No concerns | No concerns | No concerns | No concerns | No concerns | High | -- |
| Alteplase 0.6 mg/kg:Tenecteplase 0.1 mg/kg | -- | No concerns | No concerns | No concerns | Major concerns | No concerns | No concerns | Low | Imprecision |
| Alteplase 0.6 mg/kg:Tenecteplase 0.25 mg/kg | -- | No concerns | No concerns | No concerns | Major concerns | No concerns | No concerns | Low | Imprecision |
| Alteplase 0.6 mg/kg:Tenecteplase 0.32 mg/kg | -- | No concerns | No concerns | No concerns | Major concerns | No concerns | No concerns | Low | Imprecision |
| Alteplase 0.6 mg/kg:Tenecteplase 0.40 mg/kg | -- | No concerns | No concerns | No concerns | Major concerns | No concerns | No concerns | Low | Imprecision |
| Alteplase 0.6 mg/kg:rhPro-UK 35mg | -- | No concerns | No concerns | No concerns | Major concerns | No concerns | No concerns | Low | Imprecision |
| Placebo:rhPro-UK 50mg | -- | No concerns | No concerns | No concerns | Major concerns | No concerns | No concerns | Low | Imprecision |
| Placebo:Reteplase 12+12 mg | -- | No concerns | No concerns | No concerns | Major concerns | No concerns | No concerns | Low | Imprecision |
| Placebo:Reteplase 18+18 mg | -- | No concerns | No concerns | No concerns | No concerns | No concerns | No concerns | High | -- |
| Placebo:Staphylokinase 10mg | -- | No concerns | No concerns | No concerns | No concerns | No concerns | No concerns | High | -- |
| Placebo:Tenecteplase 0.1 mg/kg | -- | No concerns | No concerns | No concerns | Major concerns | No concerns | No concerns | Low | Imprecision |
| Placebo:Tenecteplase 0.25 mg/kg | -- | No concerns | No concerns | No concerns | No concerns | No concerns | No concerns | High | -- |
| Placebo:Tenecteplase 0.32 mg/kg | -- | No concerns | No concerns | No concerns | Major concerns | No concerns | No concerns | Low | Imprecision |
| Placebo:Tenecteplase 0.40 mg/kg | -- | No concerns | No concerns | No concerns | Major concerns | No concerns | No concerns | Low | Imprecision |
| Placebo:rhPro-UK 35mg | -- | No concerns | No concerns | No concerns | No concerns | No concerns | No concerns | High | -- |
| rhPro-UK 50mg:Reteplase 12+12 mg | -- | No concerns | No concerns | No concerns | Major concerns | No concerns | No concerns | Low | Imprecision |
| rhPro-UK 50mg:Reteplase 18+18 mg | -- | No concerns | No concerns | No concerns | Major concerns | No concerns | No concerns | Low | Imprecision |
| rhPro-UK 50mg:Staphylokinase 10mg | -- | No concerns | No concerns | No concerns | Major concerns | No concerns | No concerns | Low | Imprecision |
| rhPro-UK 50mg:Tenecteplase 0.1 mg/kg | -- | No concerns | No concerns | No concerns | Major concerns | No concerns | No concerns | Low | Imprecision |
| rhPro-UK 50mg:Tenecteplase 0.25 mg/kg | -- | No concerns | No concerns | No concerns | Major concerns | No concerns | No concerns | Low | Imprecision |
| rhPro-UK 50mg:Tenecteplase 0.32 mg/kg | -- | No concerns | No concerns | No concerns | Major concerns | No concerns | No concerns | Low | Imprecision |
| rhPro-UK 50mg:Tenecteplase 0.40 mg/kg | -- | No concerns | No concerns | No concerns | Major concerns | No concerns | No concerns | Low | Imprecision |
| Reteplase 12+12 mg:Staphylokinase 10mg | -- | No concerns | No concerns | No concerns | No concerns | Major concerns | No concerns | Low | Heterogeneity |
| Reteplase 12+12 mg:Tenecteplase 0.1 mg/kg | -- | No concerns | No concerns | No concerns | Major concerns | No concerns | No concerns | Low | Imprecision |
| Reteplase 12+12 mg:Tenecteplase 0.25 mg/kg | -- | No concerns | No concerns | No concerns | Major concerns | No concerns | No concerns | Low | Imprecision |
| Reteplase 12+12 mg:Tenecteplase 0.32 mg/kg | -- | No concerns | No concerns | No concerns | Major concerns | No concerns | No concerns | Low | Imprecision |
| Reteplase 12+12 mg:Tenecteplase 0.40 mg/kg | -- | No concerns | No concerns | No concerns | Major concerns | No concerns | No concerns | Low | Imprecision |
| Reteplase 12+12 mg:rhPro-UK 35mg | -- | No concerns | No concerns | No concerns | Major concerns | No concerns | No concerns | Low | Imprecision |
| Reteplase 18+18 mg:Staphylokinase 10mg | -- | No concerns | No concerns | No concerns | Major concerns | No concerns | No concerns | Low | Imprecision |
| Reteplase 18+18 mg:Tenecteplase 0.1 mg/kg | -- | No concerns | No concerns | No concerns | Major concerns | No concerns | No concerns | Low | Imprecision |
| Reteplase 18+18 mg:Tenecteplase 0.25 mg/kg | -- | No concerns | No concerns | No concerns | No concerns | No concerns | No concerns | High | -- |
| Reteplase 18+18 mg:Tenecteplase 0.32 mg/kg | -- | No concerns | No concerns | No concerns | Major concerns | No concerns | No concerns | Low | Imprecision |
| Reteplase 18+18 mg:Tenecteplase 0.40 mg/kg | -- | No concerns | No concerns | No concerns | Major concerns | No concerns | No concerns | Low | Imprecision |
| Reteplase 18+18 mg:rhPro-UK 35mg | -- | No concerns | No concerns | No concerns | No concerns | No concerns | No concerns | High | -- |
| Staphylokinase 10mg:Tenecteplase 0.1 mg/kg | -- | No concerns | No concerns | No concerns | No concerns | No concerns | No concerns | High | -- |
| Staphylokinase 10mg:Tenecteplase 0.25 mg/kg | -- | No concerns | No concerns | No concerns | No concerns | No concerns | No concerns | High | -- |
| Staphylokinase 10mg:Tenecteplase 0.32 mg/kg | -- | No concerns | No concerns | No concerns | No concerns | Major concerns | No concerns | Low | Heterogeneity |
| Staphylokinase 10mg:Tenecteplase 0.40 mg/kg | -- | No concerns | No concerns | No concerns | No concerns | No concerns | No concerns | High | -- |
| rhPro-UK 35mg:Staphylokinase 10mg | -- | No concerns | No concerns | No concerns | No concerns | No concerns | No concerns | High | -- |
| rhPro-UK 35mg:Tenecteplase 0.1 mg/kg | -- | No concerns | No concerns | No concerns | Major concerns | No concerns | No concerns | Low | Imprecision |
| rhPro-UK 35mg:Tenecteplase 0.25 mg/kg | -- | No concerns | No concerns | No concerns | Major concerns | No concerns | No concerns | Low | Imprecision |
| Tenecteplase 0.32 mg/kg:Tenecteplase 0.40 mg/kg | -- | No concerns | No concerns | No concerns | Major concerns | No concerns | No concerns | Low | Imprecision |
| rhPro-UK 35mg:Tenecteplase 0.32 mg/kg | -- | No concerns | No concerns | No concerns | Major concerns | No concerns | No concerns | Low | Imprecision |
| rhPro-UK 35mg:Tenecteplase 0.40 mg/kg | -- | No concerns | No concerns | No concerns | Major concerns | No concerns | No concerns | Low | Imprecision |

Abbreviation: rhPro-UK, recombinant human prourokinase; Staphylokinase, non-immunogenic recombinant staphylokinase

**Supplementary Table 4. CINeMA on good functional outcome at 90 days.**

| **Comparison** | **Number of studies** | **Within-study bias** | **Reporting bias** | **Indirectness** | **Imprecision** | **Heterogeneity** | **Incoherence** | **Confidence rating** | **Reason(s) for downgrading** |
| --- | --- | --- | --- | --- | --- | --- | --- | --- | --- |
| Alteplase 0.6 mg:Alteplase 0.9 mg | 1 | No concerns | Low risk | No concerns | Major concerns | No concerns | No concerns | Low | Imprecision |
| Alteplase 0.9 mg:Placebo | 2 | No concerns | Low risk | No concerns | Major concerns | No concerns | No concerns | Low | Imprecision |
| Alteplase 0.9 mg:Prourokinase 50mg | 1 | No concerns | Low risk | No concerns | Major concerns | No concerns | No concerns | Low | Imprecision |
| Alteplase 0.9 mg:Reteplase 12+12 mg | 1 | No concerns | Low risk | No concerns | Major concerns | No concerns | No concerns | Low | Imprecision |
| Alteplase 0.9 mg:Reteplase 18+18 mg | 2 | No concerns | Low risk | No concerns | No concerns | Major concerns | No concerns | Low | Heterogeneity |
| Alteplase 0.9 mg:Staphylokinase 10mg | 1 | No concerns | Low risk | No concerns | Major concerns | No concerns | No concerns | Low | Imprecision |
| Alteplase 0.9 mg:Tenecteplase 0.1 mg | 1 | No concerns | Low risk | No concerns | Major concerns | No concerns | No concerns | Low | Imprecision |
| Alteplase 0.9 mg:Tenecteplase 0.25 mg | 9 | No concerns | Low risk | No concerns | Major concerns | No concerns | No concerns | Low | Imprecision |
| Alteplase 0.9 mg:Tenecteplase 0.32 mg | 1 | No concerns | Low risk | No concerns | Major concerns | No concerns | No concerns | Low | Imprecision |
| Alteplase 0.9 mg:rhPro-UK 35mg | 3 | No concerns | Low risk | No concerns | Major concerns | No concerns | No concerns | Low | Imprecision |
| Prourokinase 50mg:rhPro-UK 35mg | 1 | No concerns | Low risk | No concerns | Major concerns | No concerns | No concerns | Low | Imprecision |
| Reteplase 12+12 mg:Reteplase 18+18 mg | 1 | No concerns | Low risk | No concerns | Major concerns | No concerns | No concerns | Low | Imprecision |
| Tenecteplase 0.1 mg:Tenecteplase 0.25 mg | 1 | No concerns | Low risk | No concerns | Major concerns | No concerns | No concerns | Low | Imprecision |
| Tenecteplase 0.1 mg:Tenecteplase 0.32 mg | 1 | No concerns | Low risk | No concerns | Major concerns | No concerns | No concerns | Low | Imprecision |
| Tenecteplase 0.25 mg:Tenecteplase 0.32 mg | 1 | No concerns | Low risk | No concerns | Major concerns | No concerns | No concerns | Low | Imprecision |
| Tenecteplase 0.25 mg:Tenecteplase 0.40 mg | 1 | No concerns | Low risk | No concerns | Major concerns | No concerns | No concerns | Low | Imprecision |
| Alteplase 0.6 mg:Placebo | -- | No concerns | Low risk | No concerns | Major concerns | No concerns | No concerns | Low | Imprecision |
| Alteplase 0.6 mg:Prourokinase 50mg | -- | No concerns | Low risk | No concerns | Major concerns | No concerns | No concerns | Low | Imprecision |
| Alteplase 0.6 mg:Reteplase 12+12 mg | -- | No concerns | Low risk | No concerns | Major concerns | No concerns | No concerns | Low | Imprecision |
| Alteplase 0.6 mg:Reteplase 18+18 mg | -- | No concerns | Low risk | No concerns | No concerns | Major concerns | No concerns | Low | Heterogeneity |
| Alteplase 0.6 mg:Staphylokinase 10mg | -- | No concerns | Low risk | No concerns | Major concerns | No concerns | No concerns | Low | Imprecision |
| Alteplase 0.6 mg:Tenecteplase 0.1 mg | -- | No concerns | Low risk | No concerns | Major concerns | No concerns | No concerns | Low | Imprecision |
| Alteplase 0.6 mg:Tenecteplase 0.25 mg | -- | No concerns | Low risk | No concerns | Major concerns | No concerns | No concerns | Low | Imprecision |
| Alteplase 0.6 mg:Tenecteplase 0.32 mg | -- | No concerns | Low risk | No concerns | Major concerns | No concerns | No concerns | Low | Imprecision |
| Alteplase 0.6 mg:Tenecteplase 0.40 mg | -- | No concerns | Low risk | No concerns | Major concerns | No concerns | No concerns | Low | Imprecision |
| Alteplase 0.6 mg:rhPro-UK 35mg | -- | No concerns | Low risk | No concerns | Major concerns | No concerns | No concerns | Low | Imprecision |
| Alteplase 0.9 mg:Tenecteplase 0.40 mg | -- | No concerns | Low risk | No concerns | Major concerns | No concerns | No concerns | Low | Imprecision |
| Placebo:Prourokinase 50mg | -- | No concerns | Low risk | No concerns | Major concerns | No concerns | No concerns | Low | Imprecision |
| Placebo:Reteplase 12+12 mg | -- | No concerns | Low risk | No concerns | Major concerns | No concerns | No concerns | Low | Imprecision |
| Placebo:Reteplase 18+18 mg | -- | No concerns | Low risk | No concerns | No concerns | Major concerns | No concerns | Low | Heterogeneity |
| Placebo:Staphylokinase 10mg | -- | No concerns | Low risk | No concerns | Major concerns | No concerns | No concerns | Low | Imprecision |
| Placebo:Tenecteplase 0.1 mg | -- | No concerns | Low risk | No concerns | Major concerns | No concerns | No concerns | Low | Imprecision |
| Placebo:Tenecteplase 0.25 mg | -- | No concerns | Low risk | No concerns | Major concerns | No concerns | No concerns | Low | Imprecision |
| Placebo:Tenecteplase 0.32 mg | -- | No concerns | Low risk | No concerns | Major concerns | No concerns | No concerns | Low | Imprecision |
| Placebo:Tenecteplase 0.40 mg | -- | No concerns | Low risk | No concerns | Major concerns | No concerns | No concerns | Low | Imprecision |
| Placebo:rhPro-UK 35mg | -- | No concerns | Low risk | No concerns | Major concerns | No concerns | No concerns | Low | Imprecision |
| Prourokinase 50mg:Reteplase 12+12 mg | -- | No concerns | Low risk | No concerns | Major concerns | No concerns | No concerns | Low | Imprecision |
| Prourokinase 50mg:Reteplase 18+18 mg | -- | No concerns | Low risk | No concerns | Major concerns | No concerns | No concerns | Low | Imprecision |
| Prourokinase 50mg:Staphylokinase 10mg | -- | No concerns | Low risk | No concerns | Major concerns | No concerns | No concerns | Low | Imprecision |
| Prourokinase 50mg:Tenecteplase 0.1 mg | -- | No concerns | Low risk | No concerns | Major concerns | No concerns | No concerns | Low | Imprecision |
| Prourokinase 50mg:Tenecteplase 0.25 mg | -- | No concerns | Low risk | No concerns | Major concerns | No concerns | No concerns | Low | Imprecision |
| Prourokinase 50mg:Tenecteplase 0.32 mg | -- | No concerns | Low risk | No concerns | Major concerns | No concerns | No concerns | Low | Imprecision |
| Prourokinase 50mg:Tenecteplase 0.40 mg | -- | No concerns | Low risk | No concerns | Major concerns | No concerns | No concerns | Low | Imprecision |
| Reteplase 12+12 mg:Staphylokinase 10mg | -- | No concerns | Low risk | No concerns | Major concerns | No concerns | No concerns | Low | Imprecision |
| Reteplase 12+12 mg:Tenecteplase 0.1 mg | -- | No concerns | Low risk | No concerns | Major concerns | No concerns | No concerns | Low | Imprecision |
| Reteplase 12+12 mg:Tenecteplase 0.25 mg | -- | No concerns | Low risk | No concerns | Major concerns | No concerns | No concerns | Low | Imprecision |
| Reteplase 12+12 mg:Tenecteplase 0.32 mg | -- | No concerns | Low risk | No concerns | Major concerns | No concerns | No concerns | Low | Imprecision |
| Reteplase 12+12 mg:Tenecteplase 0.40 mg | -- | No concerns | Low risk | No concerns | Major concerns | No concerns | No concerns | Low | Imprecision |
| Reteplase 12+12 mg:rhPro-UK 35mg | -- | No concerns | Low risk | No concerns | Major concerns | No concerns | No concerns | Low | Imprecision |
| Reteplase 18+18 mg:Staphylokinase 10mg | -- | No concerns | Low risk | No concerns | Major concerns | No concerns | No concerns | Low | Imprecision |
| Reteplase 18+18 mg:Tenecteplase 0.1 mg | -- | No concerns | Low risk | No concerns | Major concerns | No concerns | No concerns | Low | Imprecision |
| Reteplase 18+18 mg:Tenecteplase 0.25 mg | -- | No concerns | Low risk | No concerns | Major concerns | No concerns | No concerns | Low | Imprecision |
| Reteplase 18+18 mg:Tenecteplase 0.32 mg | -- | No concerns | Low risk | No concerns | Major concerns | No concerns | No concerns | Low | Imprecision |
| Reteplase 18+18 mg:Tenecteplase 0.40 mg | -- | No concerns | Low risk | No concerns | Major concerns | No concerns | No concerns | Low | Imprecision |
| Reteplase 18+18 mg:rhPro-UK 35mg | -- | No concerns | Low risk | No concerns | Major concerns | No concerns | No concerns | Low | Imprecision |
| Staphylokinase 10mg:Tenecteplase 0.1 mg | -- | No concerns | Low risk | No concerns | Major concerns | No concerns | No concerns | Low | Imprecision |
| Staphylokinase 10mg:Tenecteplase 0.25 mg | -- | No concerns | Low risk | No concerns | Major concerns | No concerns | No concerns | Low | Imprecision |
| Staphylokinase 10mg:Tenecteplase 0.32 mg | -- | No concerns | Low risk | No concerns | Major concerns | No concerns | No concerns | Low | Imprecision |
| Staphylokinase 10mg:Tenecteplase 0.40 mg | -- | No concerns | Low risk | No concerns | Major concerns | No concerns | No concerns | Low | Imprecision |
| rhPro-UK 35mg:Staphylokinase 10mg | -- | No concerns | Low risk | No concerns | Major concerns | No concerns | No concerns | Low | Imprecision |
| Tenecteplase 0.1 mg:Tenecteplase 0.40 mg | -- | No concerns | Low risk | No concerns | Major concerns | No concerns | No concerns | Low | Imprecision |
| rhPro-UK 35mg:Tenecteplase 0.1 mg | -- | No concerns | Low risk | No concerns | Major concerns | No concerns | No concerns | Low | Imprecision |
| rhPro-UK 35mg:Tenecteplase 0.25 mg | -- | No concerns | Low risk | No concerns | Major concerns | No concerns | No concerns | Low | Imprecision |
| Tenecteplase 0.32 mg:Tenecteplase 0.40 mg | -- | No concerns | Low risk | No concerns | Major concerns | No concerns | No concerns | Low | Imprecision |
| rhPro-UK 35mg:Tenecteplase 0.32 mg | -- | No concerns | Low risk | No concerns | Major concerns | No concerns | No concerns | Low | Imprecision |
| rhPro-UK 35mg:Tenecteplase 0.40 mg | -- | No concerns | Low risk | No concerns | Major concerns | No concerns | No concerns | Low | Imprecision |

Abbreviation: rhPro-UK, recombinant human prourokinase; Staphylokinase, non-immunogenic recombinant staphylokinase

**Supplementary Table 5. CINeMA on all-cause mortality at 90 days.**

| **Comparison** | **Number of studies** | **Within-study bias** | **Reporting bias** | **Indirectness** | **Imprecision** | **Heterogeneity** | **Incoherence** | **Confidence rating** | **Reason(s) for downgrading** |
| --- | --- | --- | --- | --- | --- | --- | --- | --- | --- |
| Alteplase 0.6 mg/kg:Alteplase 0.9 mg/kg | 1 | No concerns | No concerns | No concerns | Major concerns | No concerns | No concerns | Low | Imprecision |
| Alteplase 0.9 mg/kg:Placebo | 2 | No concerns | No concerns | No concerns | Major concerns | No concerns | No concerns | Low | Imprecision |
| Alteplase 0.9 mg/kg:Prourokinase 50mg | 1 | No concerns | No concerns | No concerns | Major concerns | No concerns | No concerns | Low | Imprecision |
| Alteplase 0.9 mg/kg:Reteplase 12+12 mg | 1 | No concerns | No concerns | No concerns | Major concerns | No concerns | No concerns | Low | Imprecision |
| Alteplase 0.9 mg/kg:Reteplase 18+18 mg | 2 | No concerns | No concerns | No concerns | Major concerns | No concerns | No concerns | Low | Imprecision |
| Alteplase 0.9 mg/kg:Staphylokinase 10mg | 1 | No concerns | No concerns | No concerns | Major concerns | No concerns | No concerns | Low | Imprecision |
| Alteplase 0.9 mg/kg:Tenecteplase 0.1 mg/kg | 2 | No concerns | No concerns | No concerns | Major concerns | No concerns | No concerns | Low | Imprecision |
| Alteplase 0.9 mg/kg:Tenecteplase 0.25 mg/kg | 10 | No concerns | No concerns | No concerns | Major concerns | No concerns | No concerns | Low | Imprecision |
| Alteplase 0.9 mg/kg:Tenecteplase 0.32 mg/kg | 1 | No concerns | No concerns | No concerns | Major concerns | No concerns | No concerns | Low | Imprecision |
| Alteplase 0.9 mg/kg:Tenecteplase 0.40 mg/kg | 2 | No concerns | No concerns | No concerns | Major concerns | No concerns | No concerns | Low | Imprecision |
| Alteplase 0.9 mg/kg:rhPro-UK 35mg | 3 | No concerns | No concerns | No concerns | Major concerns | No concerns | No concerns | Low | Imprecision |
| Prourokinase 50mg:rhPro-UK 35mg | 1 | No concerns | No concerns | No concerns | Major concerns | No concerns | No concerns | Low | Imprecision |
| Reteplase 12+12 mg:Reteplase 18+18 mg | 1 | No concerns | No concerns | No concerns | Major concerns | No concerns | No concerns | Low | Imprecision |
| Tenecteplase 0.1 mg/kg:Tenecteplase 0.25 mg/kg | 2 | No concerns | No concerns | No concerns | Major concerns | No concerns | No concerns | Low | Imprecision |
| Tenecteplase 0.1 mg/kg:Tenecteplase 0.32 mg/kg | 1 | No concerns | No concerns | No concerns | Major concerns | No concerns | No concerns | Low | Imprecision |
| Tenecteplase 0.1 mg/kg:Tenecteplase 0.40 mg/kg | 1 | No concerns | No concerns | No concerns | Major concerns | No concerns | No concerns | Low | Imprecision |
| Tenecteplase 0.25 mg/kg:Tenecteplase 0.32 mg/kg | 1 | No concerns | No concerns | No concerns | Major concerns | No concerns | No concerns | Low | Imprecision |
| Tenecteplase 0.25 mg/kg:Tenecteplase 0.40 mg/kg | 2 | No concerns | No concerns | No concerns | Major concerns | No concerns | No concerns | Low | Imprecision |
| Alteplase 0.6 mg/kg:Placebo | -- | No concerns | No concerns | No concerns | Major concerns | No concerns | No concerns | Low | Imprecision |
| Alteplase 0.6 mg/kg:Prourokinase 50mg | -- | No concerns | No concerns | No concerns | Major concerns | No concerns | No concerns | Low | Imprecision |
| Alteplase 0.6 mg/kg:Reteplase 12+12 mg | -- | No concerns | No concerns | No concerns | Major concerns | No concerns | No concerns | Low | Imprecision |
| Alteplase 0.6 mg/kg:Reteplase 18+18 mg | -- | No concerns | No concerns | No concerns | Major concerns | No concerns | No concerns | Low | Imprecision |
| Alteplase 0.6 mg/kg:Staphylokinase 10mg | -- | No concerns | No concerns | No concerns | Major concerns | No concerns | No concerns | Low | Imprecision |
| Alteplase 0.6 mg/kg:Tenecteplase 0.1 mg/kg | -- | No concerns | No concerns | No concerns | Major concerns | No concerns | No concerns | Low | Imprecision |
| Alteplase 0.6 mg/kg:Tenecteplase 0.25 mg/kg | -- | No concerns | No concerns | No concerns | Major concerns | No concerns | No concerns | Low | Imprecision |
| Alteplase 0.6 mg/kg:Tenecteplase 0.32 mg/kg | -- | No concerns | No concerns | No concerns | Major concerns | No concerns | No concerns | Low | Imprecision |
| Alteplase 0.6 mg/kg:Tenecteplase 0.40 mg/kg | -- | No concerns | No concerns | No concerns | Major concerns | No concerns | No concerns | Low | Imprecision |
| Alteplase 0.6 mg/kg:rhPro-UK 35mg | -- | No concerns | No concerns | No concerns | Major concerns | No concerns | No concerns | Low | Imprecision |
| Placebo:Prourokinase 50mg | -- | No concerns | No concerns | No concerns | Major concerns | No concerns | No concerns | Low | Imprecision |
| Placebo:Reteplase 12+12 mg | -- | No concerns | No concerns | No concerns | Major concerns | No concerns | No concerns | Low | Imprecision |
| Placebo:Reteplase 18+18 mg | -- | No concerns | No concerns | No concerns | Major concerns | No concerns | No concerns | Low | Imprecision |
| Placebo:Staphylokinase 10mg | -- | No concerns | No concerns | No concerns | Major concerns | No concerns | No concerns | Low | Imprecision |
| Placebo:Tenecteplase 0.1 mg/kg | -- | No concerns | No concerns | No concerns | Major concerns | No concerns | No concerns | Low | Imprecision |
| Placebo:Tenecteplase 0.25 mg/kg | -- | No concerns | No concerns | No concerns | Major concerns | No concerns | No concerns | Low | Imprecision |
| Placebo:Tenecteplase 0.32 mg/kg | -- | No concerns | No concerns | No concerns | Major concerns | No concerns | No concerns | Low | Imprecision |
| Placebo:Tenecteplase 0.40 mg/kg | -- | No concerns | No concerns | No concerns | Major concerns | No concerns | No concerns | Low | Imprecision |
| Placebo:rhPro-UK 35mg | -- | No concerns | No concerns | No concerns | Major concerns | No concerns | No concerns | Low | Imprecision |
| Prourokinase 50mg:Reteplase 12+12 mg | -- | No concerns | No concerns | No concerns | Major concerns | No concerns | No concerns | Low | Imprecision |
| Prourokinase 50mg:Reteplase 18+18 mg | -- | No concerns | No concerns | No concerns | Major concerns | No concerns | No concerns | Low | Imprecision |
| Prourokinase 50mg:Staphylokinase 10mg | -- | No concerns | No concerns | No concerns | Major concerns | No concerns | No concerns | Low | Imprecision |
| Prourokinase 50mg:Tenecteplase 0.1 mg/kg | -- | No concerns | No concerns | No concerns | Major concerns | No concerns | No concerns | Low | Imprecision |
| Prourokinase 50mg:Tenecteplase 0.25 mg/kg | -- | No concerns | No concerns | No concerns | Major concerns | No concerns | No concerns | Low | Imprecision |
| Prourokinase 50mg:Tenecteplase 0.32 mg/kg | -- | No concerns | No concerns | No concerns | Major concerns | No concerns | No concerns | Low | Imprecision |
| Prourokinase 50mg:Tenecteplase 0.40 mg/kg | -- | No concerns | No concerns | No concerns | Major concerns | No concerns | No concerns | Low | Imprecision |
| Reteplase 12+12 mg:Staphylokinase 10mg | -- | No concerns | No concerns | No concerns | Major concerns | No concerns | No concerns | Low | Imprecision |
| Reteplase 12+12 mg:Tenecteplase 0.1 mg/kg | -- | No concerns | No concerns | No concerns | Major concerns | No concerns | No concerns | Low | Imprecision |
| Reteplase 12+12 mg:Tenecteplase 0.25 mg/kg | -- | No concerns | No concerns | No concerns | Major concerns | No concerns | No concerns | Low | Imprecision |
| Reteplase 12+12 mg:Tenecteplase 0.32 mg/kg | -- | No concerns | No concerns | No concerns | Major concerns | No concerns | No concerns | Low | Imprecision |
| Reteplase 12+12 mg:Tenecteplase 0.40 mg/kg | -- | No concerns | No concerns | No concerns | Major concerns | No concerns | No concerns | Low | Imprecision |
| Reteplase 12+12 mg:rhPro-UK 35mg | -- | No concerns | No concerns | No concerns | Major concerns | No concerns | No concerns | Low | Imprecision |
| Reteplase 18+18 mg:Staphylokinase 10mg | -- | No concerns | No concerns | No concerns | Major concerns | No concerns | No concerns | Low | Imprecision |
| Reteplase 18+18 mg:Tenecteplase 0.1 mg/kg | -- | No concerns | No concerns | No concerns | Major concerns | No concerns | No concerns | Low | Imprecision |
| Reteplase 18+18 mg:Tenecteplase 0.25 mg/kg | -- | No concerns | No concerns | No concerns | Major concerns | No concerns | No concerns | Low | Imprecision |
| Reteplase 18+18 mg:Tenecteplase 0.32 mg/kg | -- | No concerns | No concerns | No concerns | Major concerns | No concerns | No concerns | Low | Imprecision |
| Reteplase 18+18 mg:Tenecteplase 0.40 mg/kg | -- | No concerns | No concerns | No concerns | Major concerns | No concerns | No concerns | Low | Imprecision |
| Reteplase 18+18 mg:rhPro-UK 35mg | -- | No concerns | No concerns | No concerns | Major concerns | No concerns | No concerns | Low | Imprecision |
| Staphylokinase 10mg:Tenecteplase 0.1 mg/kg | -- | No concerns | No concerns | No concerns | Major concerns | No concerns | No concerns | Low | Imprecision |
| Staphylokinase 10mg:Tenecteplase 0.25 mg/kg | -- | No concerns | No concerns | No concerns | Major concerns | No concerns | No concerns | Low | Imprecision |
| Staphylokinase 10mg:Tenecteplase 0.32 mg/kg | -- | No concerns | No concerns | No concerns | Major concerns | No concerns | No concerns | Low | Imprecision |
| Staphylokinase 10mg:Tenecteplase 0.40 mg/kg | -- | No concerns | No concerns | No concerns | Major concerns | No concerns | No concerns | Low | Imprecision |
| rhPro-UK 35mg:Staphylokinase 10mg | -- | No concerns | No concerns | No concerns | Major concerns | No concerns | No concerns | Low | Imprecision |
| rhPro-UK 35mg:Tenecteplase 0.1 mg/kg | -- | No concerns | No concerns | No concerns | Major concerns | No concerns | No concerns | Low | Imprecision |
| rhPro-UK 35mg:Tenecteplase 0.25 mg/kg | -- | No concerns | No concerns | No concerns | Major concerns | No concerns | No concerns | Low | Imprecision |
| Tenecteplase 0.32 mg/kg:Tenecteplase 0.40 mg/kg | -- | No concerns | No concerns | No concerns | Major concerns | No concerns | No concerns | Low | Imprecision |
| rhPro-UK 35mg:Tenecteplase 0.32 mg/kg | -- | No concerns | No concerns | No concerns | Major concerns | No concerns | No concerns | Low | Imprecision |
| rhPro-UK 35mg:Tenecteplase 0.40 mg/kg | -- | No concerns | No concerns | No concerns | Major concerns | No concerns | No concerns | Low | Imprecision |

Abbreviation: rhPro-UK, recombinant human prourokinase; Staphylokinase, non-immunogenic recombinant staphylokinase

**Supplementary Table 6. CINeMA on symptomatic intracranial hemorrhage events.**

| **Comparison** | **Number of studies** | **Within-study bias** | **Reporting bias** | **Indirectness** | **Imprecision** | **Heterogeneity** | **Incoherence** | **Confidence rating** | **Reason(s) for downgrading** |
| --- | --- | --- | --- | --- | --- | --- | --- | --- | --- |
| Alteplase 0.6 mg/kg:Alteplase 0.9 mg/kg | 1 | No concerns | Low risk | No concerns | No concerns | No concerns | No concerns | High | -- |
| Alteplase 0.9 mg/kg:Placebo | 2 | No concerns | Low risk | No concerns | No concerns | No concerns | No concerns | High | -- |
| Alteplase 0.9 mg/kg:Prourokinase 50mg | 1 | No concerns | Low risk | No concerns | Major concerns | No concerns | No concerns | Low | Imprecision |
| Alteplase 0.9 mg/kg:Reteplase 12+12 mg | 1 | No concerns | Low risk | No concerns | Major concerns | No concerns | No concerns | Low | Imprecision |
| Alteplase 0.9 mg/kg:Reteplase 18+18 mg | 2 | No concerns | Low risk | No concerns | Major concerns | No concerns | No concerns | Low | Imprecision |
| Alteplase 0.9 mg/kg:Staphylokinase 10mg | 1 | No concerns | Low risk | No concerns | Major concerns | No concerns | No concerns | Low | Imprecision |
| Alteplase 0.9 mg/kg:Tenecteplase 0.1 mg/kg | 2 | No concerns | Low risk | No concerns | Major concerns | No concerns | No concerns | Low | Imprecision |
| Alteplase 0.9 mg/kg:Tenecteplase 0.25 mg/kg | 10 | No concerns | Low risk | No concerns | Major concerns | No concerns | No concerns | Low | Imprecision |
| Alteplase 0.9 mg/kg:Tenecteplase 0.32 mg/kg | 1 | No concerns | Low risk | No concerns | Major concerns | No concerns | No concerns | Low | Imprecision |
| Alteplase 0.9 mg/kg:Tenecteplase 0.40 mg/kg | 2 | No concerns | Low risk | No concerns | Major concerns | No concerns | No concerns | Low | Imprecision |
| Alteplase 0.9 mg/kg:rhPro-UK 35mg | 3 | No concerns | Low risk | No concerns | Major concerns | No concerns | No concerns | Low | Imprecision |
| Prourokinase 50mg:rhPro-UK 35mg | 1 | No concerns | Low risk | No concerns | Major concerns | No concerns | No concerns | Low | Imprecision |
| Reteplase 12+12 mg:Reteplase 18+18 mg | 1 | No concerns | Low risk | No concerns | Major concerns | No concerns | No concerns | Low | Imprecision |
| Tenecteplase 0.1 mg/kg:Tenecteplase 0.25 mg/kg | 2 | No concerns | Low risk | No concerns | Major concerns | No concerns | No concerns | Low | Imprecision |
| Tenecteplase 0.1 mg/kg:Tenecteplase 0.32 mg/kg | 1 | No concerns | Low risk | No concerns | Major concerns | No concerns | No concerns | Low | Imprecision |
| Tenecteplase 0.1 mg/kg:Tenecteplase 0.40 mg/kg | 1 | No concerns | Low risk | No concerns | Major concerns | No concerns | No concerns | Low | Imprecision |
| Tenecteplase 0.25 mg/kg:Tenecteplase 0.32 mg/kg | 1 | No concerns | Low risk | No concerns | Major concerns | No concerns | No concerns | Low | Imprecision |
| Tenecteplase 0.25 mg/kg:Tenecteplase 0.40 mg/kg | 2 | No concerns | Low risk | No concerns | Major concerns | No concerns | No concerns | Low | Imprecision |
| Alteplase 0.6 mg/kg:Placebo | -- | No concerns | Low risk | No concerns | Major concerns | No concerns | No concerns | Low | Imprecision |
| Alteplase 0.6 mg/kg:Prourokinase 50mg | -- | No concerns | Low risk | No concerns | Major concerns | No concerns | No concerns | Low | Imprecision |
| Alteplase 0.6 mg/kg:Reteplase 12+12 mg | -- | No concerns | Low risk | No concerns | Major concerns | No concerns | No concerns | Low | Imprecision |
| Alteplase 0.6 mg/kg:Reteplase 18+18 mg | -- | No concerns | Low risk | No concerns | Major concerns | No concerns | No concerns | Low | Imprecision |
| Alteplase 0.6 mg/kg:Staphylokinase 10mg | -- | No concerns | Low risk | No concerns | Major concerns | No concerns | No concerns | Low | Imprecision |
| Alteplase 0.6 mg/kg:Tenecteplase 0.1 mg/kg | -- | No concerns | Low risk | No concerns | Major concerns | No concerns | No concerns | Low | Imprecision |
| Alteplase 0.6 mg/kg:Tenecteplase 0.25 mg/kg | -- | No concerns | Low risk | No concerns | No concerns | No concerns | No concerns | High | -- |
| Alteplase 0.6 mg/kg:Tenecteplase 0.32 mg/kg | -- | No concerns | Low risk | No concerns | Major concerns | No concerns | No concerns | Low | Imprecision |
| Alteplase 0.6 mg/kg:Tenecteplase 0.40 mg/kg | -- | No concerns | Low risk | No concerns | No concerns | No concerns | No concerns | High | -- |
| Alteplase 0.6 mg/kg:rhPro-UK 35mg | -- | No concerns | Low risk | No concerns | Major concerns | No concerns | No concerns | Low | Imprecision |
| Placebo:Prourokinase 50mg | -- | No concerns | Low risk | No concerns | Major concerns | No concerns | No concerns | Low | Imprecision |
| Placebo:Reteplase 12+12 mg | -- | No concerns | Low risk | No concerns | No concerns | No concerns | No concerns | High | -- |
| Placebo:Reteplase 18+18 mg | -- | No concerns | Low risk | No concerns | No concerns | No concerns | No concerns | High | -- |
| Placebo:Staphylokinase 10mg | -- | No concerns | Low risk | No concerns | Major concerns | No concerns | No concerns | Low | Imprecision |
| Placebo:Tenecteplase 0.1 mg/kg | -- | No concerns | Low risk | No concerns | No concerns | No concerns | No concerns | High | -- |
| Placebo:Tenecteplase 0.25 mg/kg | -- | No concerns | Low risk | No concerns | No concerns | No concerns | No concerns | High | -- |
| Placebo:Tenecteplase 0.32 mg/kg | -- | No concerns | Low risk | No concerns | No concerns | Major concerns | No concerns | Low | Heterogeneity |
| Placebo:Tenecteplase 0.40 mg/kg | -- | No concerns | Low risk | No concerns | No concerns | No concerns | No concerns | High | -- |
| Placebo:rhPro-UK 35mg | -- | No concerns | Low risk | No concerns | Major concerns | No concerns | No concerns | Low | Imprecision |
| Prourokinase 50mg:Reteplase 12+12 mg | -- | No concerns | Low risk | No concerns | Major concerns | No concerns | No concerns | Low | Imprecision |
| Prourokinase 50mg:Reteplase 18+18 mg | -- | No concerns | Low risk | No concerns | Major concerns | No concerns | No concerns | Low | Imprecision |
| Prourokinase 50mg:Staphylokinase 10mg | -- | No concerns | Low risk | No concerns | Major concerns | No concerns | No concerns | Low | Imprecision |
| Prourokinase 50mg:Tenecteplase 0.1 mg/kg | -- | No concerns | Low risk | No concerns | Major concerns | No concerns | No concerns | Low | Imprecision |
| Prourokinase 50mg:Tenecteplase 0.25 mg/kg | -- | No concerns | Low risk | No concerns | Major concerns | No concerns | No concerns | Low | Imprecision |
| Prourokinase 50mg:Tenecteplase 0.32 mg/kg | -- | No concerns | Low risk | No concerns | Major concerns | No concerns | No concerns | Low | Imprecision |
| Prourokinase 50mg:Tenecteplase 0.40 mg/kg | -- | No concerns | Low risk | No concerns | Major concerns | No concerns | No concerns | Low | Imprecision |
| Reteplase 12+12 mg:Staphylokinase 10mg | -- | No concerns | Low risk | No concerns | No concerns | Major concerns | No concerns | Low | Heterogeneity |
| Reteplase 12+12 mg:Tenecteplase 0.1 mg/kg | -- | No concerns | Low risk | No concerns | Major concerns | No concerns | No concerns | Low | Imprecision |
| Reteplase 12+12 mg:Tenecteplase 0.25 mg/kg | -- | No concerns | Low risk | No concerns | Major concerns | No concerns | No concerns | Low | Imprecision |
| Reteplase 12+12 mg:Tenecteplase 0.32 mg/kg | -- | No concerns | Low risk | No concerns | Major concerns | No concerns | No concerns | Low | Imprecision |
| Reteplase 12+12 mg:Tenecteplase 0.40 mg/kg | -- | No concerns | Low risk | No concerns | Major concerns | No concerns | No concerns | Low | Imprecision |
| Reteplase 12+12 mg:rhPro-UK 35mg | -- | No concerns | Low risk | No concerns | Major concerns | No concerns | No concerns | Low | Imprecision |
| Reteplase 18+18 mg:Staphylokinase 10mg | -- | No concerns | Low risk | No concerns | Major concerns | No concerns | No concerns | Low | Imprecision |
| Reteplase 18+18 mg:Tenecteplase 0.1 mg/kg | -- | No concerns | Low risk | No concerns | Major concerns | No concerns | No concerns | Low | Imprecision |
| Reteplase 18+18 mg:Tenecteplase 0.25 mg/kg | -- | No concerns | Low risk | No concerns | Major concerns | No concerns | No concerns | Low | Imprecision |
| Reteplase 18+18 mg:Tenecteplase 0.32 mg/kg | -- | No concerns | Low risk | No concerns | Major concerns | No concerns | No concerns | Low | Imprecision |
| Reteplase 18+18 mg:Tenecteplase 0.40 mg/kg | -- | No concerns | Low risk | No concerns | Major concerns | No concerns | No concerns | Low | Imprecision |
| Reteplase 18+18 mg:rhPro-UK 35mg | -- | No concerns | Low risk | No concerns | Major concerns | No concerns | No concerns | Low | Imprecision |
| Staphylokinase 10mg:Tenecteplase 0.1 mg/kg | -- | No concerns | Low risk | No concerns | Major concerns | No concerns | No concerns | Low | Imprecision |
| Staphylokinase 10mg:Tenecteplase 0.25 mg/kg | -- | No concerns | Low risk | No concerns | No concerns | Major concerns | No concerns | Low | Heterogeneity |
| Staphylokinase 10mg:Tenecteplase 0.32 mg/kg | -- | No concerns | Low risk | No concerns | Major concerns | No concerns | No concerns | Low | Imprecision |
| Staphylokinase 10mg:Tenecteplase 0.40 mg/kg | -- | No concerns | Low risk | No concerns | No concerns | No concerns | No concerns | High | -- |
| rhPro-UK 35mg:Staphylokinase 10mg | -- | No concerns | Low risk | No concerns | Major concerns | No concerns | No concerns | Low | Imprecision |
| rhPro-UK 35mg:Tenecteplase 0.1 mg/kg | -- | No concerns | Low risk | No concerns | Major concerns | No concerns | No concerns | Low | Imprecision |
| rhPro-UK 35mg:Tenecteplase 0.25 mg/kg | -- | No concerns | Low risk | No concerns | Major concerns | No concerns | No concerns | Low | Imprecision |
| Tenecteplase 0.32 mg/kg:Tenecteplase 0.40 mg/kg | -- | No concerns | Low risk | No concerns | Major concerns | No concerns | No concerns | Low | Imprecision |
| rhPro-UK 35mg:Tenecteplase 0.32 mg/kg | -- | No concerns | Low risk | No concerns | Major concerns | No concerns | No concerns | Low | Imprecision |
| rhPro-UK 35mg:Tenecteplase 0.40 mg/kg | -- | No concerns | Low risk | No concerns | No concerns | No concerns | No concerns | High | -- |

Abbreviation: rhPro-UK, recombinant human prourokinase; Staphylokinase, non-immunogenic recombinant staphylokinase

**Supplementary Table 7. League diagram of 90-day mortality events.**

| Alteplase  0.6 mg/kg | 1.25 (0.99,1.58) | 0.89 (0.34,2.29) | 1.24 (0.93,1.65) | 1.01 (0.31,3.29) | 1.44 (0.81,2.58) | 1.30 (0.30,5.64) | 1.49 (0.84,2.63) | 1.28 (0.77,2.13) | 1.15 (0.26,5.15) | 0.84  (0.42,1.70) | 1.46 (0.92,2.34) |
| --- | --- | --- | --- | --- | --- | --- | --- | --- | --- | --- | --- |
| 0.80 (0.63,1.01) | Alteplase  0.9 mg/kg | 0.71 (0.28,1.78) | 1.00 (0.84,1.17) | 0.81 (0.25,2.58) | 1.16 (0.68,1.97) | 1.05 (0.25,4.44) | 1.20 (0.71,2.01) | 1.03 (0.66,1.62) | 0.93 (0.21,4.06) | 0.68  (0.35,1.31) | 1.17 (0.78,1.76) |
| 1.13 (0.44,2.91) | 1.40 (0.56,3.52) | Tenecteplase 0.1 mg/kg | 1.40 (0.55,3.52) | 1.14 (0.35,3.72) | 1.63 (0.58,4.60) | 1.47 (0.26,8.15) | 1.68 (0.58,4.82) | 1.45 (0.52,4.02) | 1.30 (0.23,7.41) | 0.95  (0.31,2.94) | 1.65 (0.60,4.50) |
| 0.81 (0.61,1.07) | 1.00 (0.85,1.18) | 0.72 (0.28,1.80) | Tenecteplase 0.25 mg/kg | 0.81 (0.25,2.60) | 1.16 (0.69,1.95) | 1.05 (0.25,4.51) | 1.20 (0.70,2.07) | 1.03 (0.64,1.67) | 0.93 (0.21,4.11) | 0.68  (0.34,1.34) | 1.18 (0.76,1.82) |
| 0.99 (0.30,3.24) | 1.24 (0.39,3.94) | 0.88 (0.27,2.89) | 1.23 (0.38,3.95) | Tenecteplase 0.32 mg/kg | 1.43 (0.40,5.08) | 1.29 (0.20,8.25) | 1.48 (0.42,5.26) | 1.27 (0.37,4.42) | 1.15 (0.18,7.49) | 0.84  (0.22,3.17) | 1.45 (0.43,4.95) |
| 0.69 (0.39,1.24) | 0.86 (0.51,1.47) | 0.61 (0.22,1.74) | 0.86 (0.51,1.44) | 0.70 (0.20,2.47) | Tenecteplase 0.40 mg/kg | 0.90 (0.19,4.22) | 1.03 (0.49,2.17) | 0.89 (0.44,1.78) | 0.80 (0.17,3.84) | 0.58  (0.25,1.36) | 1.01 (0.52,1.98) |
| 0.77 (0.18,3.32) | 0.96 (0.23,4.06) | 0.68 (0.12,3.78) | 0.95 (0.22,4.08) | 0.77 (0.12,4.93) | 1.11 (0.24,5.17) | Reteplase 12+12 mg | 1.14 (0.27,4.85) | 0.98 (0.22,4.48) | 0.89 (0.11,7.00) | 0.65  (0.13,3.17) | 1.12 (0.25,5.04) |
| 0.67 (0.38,1.19) | 0.84 (0.50,1.41) | 0.60 (0.21,1.71) | 0.83 (0.48,1.44) | 0.68 (0.19,2.41) | 0.97 (0.46,2.04) | 0.87 (0.21,3.71) | Reteplase 18+18 mg | 0.86 (0.43,1.71) | 0.78 (0.16,3.71) | 0.57  (0.24,1.31) | 0.98 (0.51,1.90) |
| 0.78 (0.47,1.30) | 0.97 (0.62,1.52) | 0.69 (0.25,1.92) | 0.97 (0.60,1.56) | 0.79 (0.23,2.72) | 1.12 (0.56,2.26) | 1.02 (0.22,4.62) | 1.16 (0.58,2.31) | rhPro-UK 35mg | 0.90 (0.21,3.86) | 0.66  (0.29,1.46) | 1.14 (0.62,2.09) |
| 0.87 (0.19,3.86) | 1.08 (0.25,4.73) | 0.77 (0.13,4.38) | 1.07 (0.24,4.75) | 0.87 (0.13,5.70) | 1.25 (0.26,6.01) | 1.13 (0.14,8.92) | 1.29 (0.27,6.17) | 1.11 (0.26,4.77) | rhPro-UK 50mg | 0.73  (0.14,3.68) | 1.27 (0.27,5.86) |
| 1.19 (0.59,2.40) | 1.48 (0.76,2.87) | 1.05 (0.34,3.27) | 1.47 (0.74,2.91) | 1.20 (0.32,4.54) | 1.71 (0.73,4.01) | 1.55 (0.32,7.60) | 1.77 (0.76,4.10) | 1.52 (0.68,3.39) | 1.37 (0.27,6.92) | Staphylokinase 10mg | 1.74 (0.80,3.77) |
| 0.68 (0.43,1.09) | 0.85 (0.57,1.28) | 0.61 (0.22,1.66) | 0.85 (0.55,1.31) | 0.69 (0.20,2.35) | 0.99 (0.51,1.92) | 0.89 (0.20,4.00) | 1.02 (0.53,1.97) | 0.88 (0.48,1.61) | 0.79 (0.17,3.65) | 0.58  (0.27,1.25) | Placebo |

Abbreviation: rhPro-UK, Recombinant human prourokinase; Staphylokinase, Non-immunogenic recombinant staphylokinase

**Supplementary Table 8. Rank of different doses of thrombolytics on 90-day mortality events.**

| **Treatment** | **SUCRA** | **PrBest** | **MeanRank** |
| --- | --- | --- | --- |
| Alteplase 0.6 mg/kg | 28.1 | 0.0 | 8.9 |
| Alteplase 0.9 mg/kg | 54.7 | 0.0 | 6.0 |
| Tenecteplase 0.1 mg/kg | 30.1 | 3.1 | 8.7 |
| Tenecteplase 0.25 mg/kg | 53.5 | 0.3 | 6.1 |
| Tenecteplase 0.32 mg/kg | 40.1 | 10.1 | 7.6 |
| Tenecteplase 0.40 mg/kg | 67.7 | 12.2 | 4.6 |
| Reteplase 12+12 mg | 54.6 | 25.4 | 6.0 |
| Reteplase 18+18 mg | 70.8 | 12.6 | 4.2 |
| rhPro-UK 35mg | 57.3 | 3.6 | 5.7 |
| rhPro-UK 50mg | 48.5 | 20.6 | 6.7 |
| Staphylokinase 10mg | 23.3 | 0.8 | 9.4 |
| Placebo | 71.3 | 11.1 | 4.2 |

Abbreviation: SUCRA, surface under the cumulative ranking; PrBest, probability of being the best; rhPro-UK, Recombinant human prourokinase; Staphylokinase, Non-immunogenic recombinant staphylokinase

**Supplementary Table 9. League diagram of symptomatic intracranial hemorrhage events.**

| Alteplase  0.6 mg/kg | 2.10 (1.17,3.76) | 3.19 (0.63,16.19) | 2.44 (1.27,4.69) | 2.97 (0.45,19.71) | 4.16 (1.53,11.26) | 6.70 (0.95,47.13) | 2.42 (0.89,6.57) | 1.13 (0.40,3.19) | 4.62 (0.32,66.10) | 0.77  (0.23,2.56) | 0.29 (0.07,1.10) |
| --- | --- | --- | --- | --- | --- | --- | --- | --- | --- | --- | --- |
| 0.48 (0.27,0.86) | Alteplase  0.9 mg/kg | 1.52 (0.33,6.93) | 1.16 (0.86,1.56) | 1.42 (0.23,8.57) | 1.98 (0.88,4.45) | 3.19 (0.50,20.57) | 1.16 (0.51,2.60) | 0.54 (0.23,1.27) | 2.20 (0.16,29.56) | 0.37  (0.13,1.05) | 0.14 (0.04,0.46) |
| 0.31 (0.06,1.59) | 0.66 (0.14,2.99) | Tenecteplase 0.1 mg/kg | 0.76 (0.17,3.51) | 0.93 (0.18,4.74) | 1.30 (0.25,6.88) | 2.10 (0.19,23.14) | 0.76 (0.14,4.23) | 0.35 (0.06,2.02) | 1.45 (0.07,29.24) | 0.24  (0.04,1.52) | 0.09 (0.01,0.63) |
| 0.41 (0.21,0.79) | 0.86 (0.64,1.16) | 1.31 (0.29,6.02) | Tenecteplase 0.25 mg/kg | 1.22 (0.20,7.45) | 1.71 (0.75,3.86) | 2.75 (0.42,18.11) | 0.99 (0.42,2.35) | 0.46 (0.19,1.15) | 1.90 (0.14,25.85) | 0.31  (0.11,0.94) | 0.12 (0.03,0.41) |
| 0.34 (0.05,2.23) | 0.71 (0.12,4.27) | 1.07 (0.21,5.47) | 0.82 (0.13,5.01) | Tenecteplase 0.32 mg/kg | 1.40 (0.20,9.77) | 2.25 (0.17,30.06) | 0.82 (0.11,5.87) | 0.38 (0.05,2.80) | 1.56 (0.07,36.64) | 0.26  (0.03,2.08) | 0.10 (0.01,0.85) |
| 0.24 (0.09,0.65) | 0.50 (0.22,1.13) | 0.77 (0.15,4.06) | 0.59 (0.26,1.33) | 0.71 (0.10,4.99) | Tenecteplase 0.40 mg/kg | 1.61 (0.21,12.26) | 0.58 (0.19,1.83) | 0.27 (0.08,0.88) | 1.11 (0.07,16.85) | 0.18  (0.05,0.70) | 0.07 (0.02,0.30) |
| 0.15 (0.02,1.05) | 0.31 (0.05,2.02) | 0.48 (0.04,5.26) | 0.36 (0.06,2.40) | 0.44 (0.03,5.91) | 0.62 (0.08,4.73) | Reteplase 12+12 mg | 0.36 (0.06,2.33) | 0.17 (0.02,1.31) | 0.69 (0.03,16.84) | 0.11  (0.01,0.97) | 0.04 (0.00,0.39) |
| 0.41 (0.15,1.12) | 0.87 (0.39,1.94) | 1.32 (0.24,7.34) | 1.01 (0.42,2.38) | 1.23 (0.17,8.83) | 1.72 (0.55,5.39) | 2.76 (0.43,17.79) | Reteplase 18+18 mg | 0.47 (0.14,1.52) | 1.91 (0.13,28.93) | 0.32  (0.08,1.20) | 0.12 (0.03,0.51) |
| 0.88 (0.31,2.49) | 1.85 (0.79,4.37) | 2.82 (0.49,16.09) | 2.15 (0.87,5.34) | 2.62 (0.36,19.28) | 3.67 (1.13,11.93) | 5.92 (0.76,45.97) | 2.14 (0.66,6.96) | rhPro-UK 35mg | 4.08 (0.30,54.75) | 0.68  (0.17,2.64) | 0.25 (0.06,1.12) |
| 0.22 (0.02,3.10) | 0.45 (0.03,6.09) | 0.69 (0.03,13.96) | 0.53 (0.04,7.20) | 0.64 (0.03,15.14) | 0.90 (0.06,13.65) | 1.45 (0.06,35.38) | 0.52 (0.03,7.96) | 0.24 (0.02,3.29) | rhPro-UK 50mg | 0.17  (0.01,2.73) | 0.06 (0.00,1.09) |
| 1.30 (0.39,4.35) | 2.73 (0.95,7.85) | 4.16 (0.66,26.38) | 3.18 (1.06,9.51) | 3.87 (0.48,31.21) | 5.42 (1.44,20.47) | 8.74 (1.03,74.25) | 3.16 (0.84,11.94) | 1.48 (0.38,5.75) | 6.03 (0.37,99.31) | Staphylokinase 10mg | 0.37 (0.07,1.87) |
| 3.50 (0.91,13.54) | 7.35 (2.17,24.87) | 11.19 (1.60,78.23) | 8.54 (2.43,29.96) | 10.41 (1.18,91.55) | 14.57 (3.37,62.90) | 23.47 (2.53,217.35) | 8.49 (1.96,36.69) | 3.97 (0.89,17.61) | 16.19 (0.92,285.01) | 2.69 (0.54,13.47) | Placebo |

Abbreviation: rhPro-UK, Recombinant human prourokinase; Staphylokinase, Non-immunogenic recombinant staphylokinase

**Supplementary Table 10. Rank of different doses of thrombolytics on symptomatic intracranial hemorrhage events.**

| **Treatment** | **SUCRA** | **PrBest** | **MeanRank** |
| --- | --- | --- | --- |
| Alteplase 0.6 mg/kg | 22.3 | 0.0 | 9.5 |
| Alteplase 0.9 mg/kg | 49.8 | 0.0 | 6.5 |
| Tenecteplase 0.1 mg/kg | 65.9 | 8.2 | 4.7 |
| Tenecteplase 0.25 mg/kg | 60.1 | 0.1 | 5.4 |
| Tenecteplase 0.32 mg/kg | 62.6 | 10 | 5.1 |
| Tenecteplase 0.40 mg/kg | 79.4 | 9.6 | 3.3 |
| Reteplase 12+12 mg | 83.8 | 40.7 | 2.8 |
| Reteplase 18+18 mg | 58.0 | 0.7 | 5.6 |
| rhPro-UK 35mg | 27.2 | 0.0 | 9.0 |
| rhPro-UK 50mg | 71.3 | 30.6 | 4.2 |
| Staphylokinase 10mg | 17.3 | 0.0 | 10.1 |
| Placebo | 2.2 | 0.0 | 11.8 |

Abbreviation: SUCRA, surface under the cumulative ranking; PrBest, probability of being the best; rhPro-UK, recombinant human prourokinase; Staphylokinase, non-immunogenic recombinant staphylokinase

**Supplementary Table 11. Network sensitive-analysis of league diagram of excellent functional outcome at 90 days.**

| Alteplase  0.6 mg/kg | 1.09 (0.95,1.25) | 1.01 (0.60,1.71) | 1.18 (1.00,1.39) | 1.08 (0.57,2.04) | 1.16 (0.81,1.68) | 1.02 (0.52,2.01) | 1.74 (1.33,2.28) | 1.23 (0.98,1.53) | 1.35 (0.60,3.02) | 2.43 (1.52,3.87) | 0.78 (0.59,1.04) |
| --- | --- | --- | --- | --- | --- | --- | --- | --- | --- | --- | --- |
| 0.92 (0.80,1.06) | Alteplase  0.9 mg/kg | 0.93 (0.56,1.55) | 1.08 (0.98,1.19) | 0.99 (0.53,1.85) | 1.07 (0.76,1.50) | 0.94 (0.48,1.82) | 1.60 (1.27,2.02) | 1.13 (0.94,1.34) | 1.24 (0.56,2.74) | 2.23 (1.43,3.48) | 0.72 (0.56,0.92) |
| 0.99 (0.58,1.67) | 1.07 (0.65,1.78) | Tenecteplase 0.1 mg/kg | 1.16 (0.70,1.92) | 1.07 (0.54,2.10) | 1.15 (0.64,2.07) | 1.01 (0.44,2.32) | 1.72 (0.98,2.99) | 1.21 (0.71,2.07) | 1.33 (0.52,3.41) | 2.39 (1.22,4.70) | 0.77 (0.44,1.36) |
| 0.85 (0.72,1.00) | 0.92 (0.84,1.02) | 0.86 (0.52,1.43) | Tenecteplase 0.25 mg/kg | 0.92 (0.49,1.71) | 0.99 (0.71,1.38) | 0.87 (0.44,1.70) | 1.48 (1.15,1.90) | 1.04 (0.85,1.27) | 1.15 (0.51,2.55) | 2.06 (1.31,3.25) | 0.67 (0.51,0.87) |
| 0.93 (0.49,1.75) | 1.01 (0.54,1.87) | 0.94 (0.48,1.85) | 1.09 (0.58,2.03) | Tenecteplase 0.32 mg/kg | 1.08 (0.53,2.17) | 0.94 (0.38,2.35) | 1.61 (0.83,3.13) | 1.13 (0.59,2.16) | 1.25 (0.45,3.42) | 2.25 (1.05,4.83) | 0.73 (0.37,1.42) |
| 0.86 (0.60,1.24) | 0.94 (0.67,1.31) | 0.87 (0.48,1.57) | 1.01 (0.72,1.42) | 0.93 (0.46,1.87) | Tenecteplase 0.40 mg/kg | 0.88 (0.42,1.85) | 1.50 (0.99,2.26) | 1.05 (0.72,1.55) | 1.16 (0.49,2.75) | 2.09 (1.19,3.65) | 0.67 (0.44,1.03) |
| 0.98 (0.50,1.93) | 1.07 (0.55,2.07) | 0.99 (0.43,2.29) | 1.15 (0.59,2.26) | 1.06 (0.43,2.63) | 1.14 (0.54,2.40) | Reteplase 12+12 mg | 1.70 (0.88,3.30) | 1.20 (0.60,2.39) | 1.32 (0.47,3.72) | 2.38 (1.07,5.30) | 0.77 (0.38,1.56) |
| 0.57 (0.44,0.75) | 0.63 (0.50,0.79) | 0.58 (0.33,1.02) | 0.68 (0.53,0.87) | 0.62 (0.32,1.21) | 0.67 (0.44,1.01) | 0.59 (0.30,1.14) | Reteplase 18+18 mg | 0.70 (0.53,0.94) | 0.77 (0.34,1.77) | 1.40 (0.84,2.31) | 0.45 (0.32,0.63) |
| 0.82 (0.65,1.02) | 0.89 (0.74,1.06) | 0.83 (0.48,1.41) | 0.96 (0.79,1.17) | 0.88 (0.46,1.68) | 0.95 (0.65,1.39) | 0.83 (0.42,1.66) | 1.42 (1.06,1.90) | rhPro-UK 35mg | 1.10 (0.50,2.43) | 1.98 (1.23,3.20) | 0.64 (0.47,0.87) |
| 0.74 (0.33,1.66) | 0.81 (0.36,1.79) | 0.75 (0.29,1.93) | 0.87 (0.39,1.94) | 0.80 (0.29,2.20) | 0.86 (0.36,2.05) | 0.76 (0.27,2.13) | 1.29 (0.56,2.95) | 0.91 (0.41,2.01) | rhPro-UK 50mg | 1.80 (0.72,4.48) | 0.58 (0.25,1.34) |
| 0.41 (0.26,0.66) | 0.45 (0.29,0.70) | 0.42 (0.21,0.82) | 0.48 (0.31,0.76) | 0.45 (0.21,0.96) | 0.48 (0.27,0.84) | 0.42 (0.19,0.94) | 0.72 (0.43,1.18) | 0.51 (0.31,0.82) | 0.56 (0.22,1.38) | Staphylokinase 10mg | 0.32 (0.19,0.54) |
| 1.28 (0.96,1.69) | 1.39 (1.09,1.77) | 1.29 (0.74,2.27) | 1.50 (1.15,1.95) | 1.38 (0.71,2.69) | 1.48 (0.98,2.26) | 1.30 (0.64,2.65) | 2.22 (1.58,3.11) | 1.56 (1.16,2.12) | 1.72 (0.75,3.95) | 3.10 (1.86,5.15) | Placebo |

Abbreviation: rhPro-UK, Recombinant human prourokinase; Staphylokinase, Non-immunogenic recombinant staphylokinase

**Supplementary Table 12. Network sensitive-analysis of league diagram of good functional outcome at 90 days.**

| Alteplase  0.6 mg | 1.03 (0.71,1.49) | 0.81 (0.35,1.87) | 1.03 (0.67,1.57) | 0.81 (0.35,1.87) | 1.15 (0.56,2.34) | 0.74 (0.30,1.81) | 1.39 (0.80,2.43) | 1.04 (0.64,1.69) | 0.80 (0.31,2.10) | 0.99 (0.59,1.67) | 0.87 (0.44,1.73) |
| --- | --- | --- | --- | --- | --- | --- | --- | --- | --- | --- | --- |
| 0.97 (0.67,1.41) | Alteplase  0.9 mg | 0.79 (0.37,1.67) | 1.00 (0.82,1.22) | 0.79 (0.37,1.67) | 1.12 (0.61,2.05) | 0.72 (0.32,1.62) | 1.35 (0.89,2.05) | 1.01 (0.74,1.38) | 0.78 (0.32,1.90) | 0.97 (0.68,1.39) | 0.85 (0.48,1.51) |
| 1.24 (0.53,2.86) | 1.27 (0.60,2.69) | Tenecteplase 0.1 mg | 1.27 (0.60,2.69) | 1.00 (0.43,2.32) | 1.42 (0.55,3.65) | 0.92 (0.31,2.76) | 1.72 (0.73,4.03) | 1.29 (0.57,2.89) | 0.99 (0.31,3.17) | 1.23 (0.53,2.84) | 1.08 (0.42,2.78) |
| 0.97 (0.64,1.48) | 1.00 (0.82,1.22) | 0.79 (0.37,1.67) | Tenecteplase 0.25 mg | 0.79 (0.37,1.67) | 1.12 (0.63,1.98) | 0.72 (0.32,1.65) | 1.35 (0.87,2.09) | 1.01 (0.71,1.45) | 0.78 (0.31,1.93) | 0.97 (0.63,1.48) | 0.85 (0.47,1.55) |
| 1.24 (0.53,2.86) | 1.27 (0.60,2.69) | 1.00 (0.43,2.32) | 1.27 (0.60,2.69) | Tenecteplase 0.32 mg | 1.42 (0.55,3.65) | 0.92 (0.31,2.76) | 1.72 (0.73,4.03) | 1.29 (0.57,2.89) | 0.99 (0.31,3.17) | 1.23 (0.53,2.84) | 1.08 (0.42,2.78) |
| 0.87 (0.43,1.78) | 0.90 (0.49,1.64) | 0.71 (0.27,1.81) | 0.90 (0.51,1.59) | 0.71 (0.27,1.81) | Tenecteplase 0.40 mg | 0.65 (0.24,1.77) | 1.21 (0.59,2.49) | 0.91 (0.46,1.79) | 0.70 (0.24,2.05) | 0.87 (0.42,1.77) | 0.76 (0.33,1.75) |
| 1.35 (0.55,3.28) | 1.38 (0.62,3.11) | 1.09 (0.36,3.27) | 1.39 (0.61,3.16) | 1.09 (0.36,3.27) | 1.54 (0.57,4.22) | Reteplase 12+12 mg | 1.87 (0.85,4.13) | 1.40 (0.59,3.33) | 1.08 (0.32,3.59) | 1.34 (0.55,3.27) | 1.18 (0.44,3.17) |
| 0.72 (0.41,1.26) | 0.74 (0.49,1.12) | 0.58 (0.25,1.36) | 0.74 (0.48,1.15) | 0.58 (0.25,1.36) | 0.83 (0.40,1.70) | 0.53 (0.24,1.18) | Reteplase 18+18 mg | 0.75 (0.45,1.25) | 0.58 (0.22,1.54) | 0.72 (0.41,1.26) | 0.63 (0.31,1.27) |
| 0.96 (0.59,1.56) | 0.99 (0.72,1.34) | 0.78 (0.35,1.75) | 0.99 (0.69,1.41) | 0.78 (0.35,1.75) | 1.10 (0.56,2.17) | 0.71 (0.30,1.69) | 1.33 (0.80,2.23) | rhPro-UK 35mg | 0.77 (0.32,1.87) | 0.96 (0.59,1.54) | 0.84 (0.44,1.61) |
| 1.25 (0.48,3.28) | 1.28 (0.53,3.12) | 1.01 (0.32,3.23) | 1.28 (0.52,3.19) | 1.01 (0.32,3.23) | 1.43 (0.49,4.20) | 0.93 (0.28,3.08) | 1.73 (0.65,4.62) | 1.30 (0.53,3.16) | rhPro-UK 50mg | 1.24 (0.48,3.25) | 1.09 (0.38,3.14) |
| 1.01 (0.60,1.69) | 1.03 (0.72,1.48) | 0.81 (0.35,1.88) | 1.03 (0.67,1.59) | 0.81 (0.35,1.88) | 1.15 (0.56,2.36) | 0.75 (0.31,1.82) | 1.40 (0.80,2.45) | 1.05 (0.65,1.69) | 0.81 (0.31,2.10) | Staphylokinase 10mg | 0.88 (0.45,1.73) |
| 1.14 (0.58,2.26) | 1.17 (0.66,2.08) | 0.92 (0.36,2.37) | 1.18 (0.64,2.15) | 0.92 (0.36,2.37) | 1.31 (0.57,3.01) | 0.85 (0.32,2.28) | 1.59 (0.78,3.21) | 1.19 (0.62,2.28) | 0.92 (0.32,2.63) | 1.14 (0.58,2.23) | Placebo |

Abbreviation: rhPro-UK, Recombinant human prourokinase; Staphylokinase, Non-immunogenic recombinant staphylokinase

**Supplementary Table 13. Network sensitive-analysis of league diagram of all-cause mortality at 90 days.**

| Alteplase  0.6 mg/kg | 1.25 (0.90,1.72) | 0.89 (0.33,2.39) | 1.27 (0.85,1.89) | 1.02 (0.30,3.44) | 1.46 (0.77,2.80) | 1.30 (0.29,5.81) | 1.48 (0.78,2.82) | 1.31 (0.73,2.35) | 1.17 (0.25,5.40) | 0.84 (0.39,1.82) | 1.47 (0.85,2.52) |
| --- | --- | --- | --- | --- | --- | --- | --- | --- | --- | --- | --- |
| 0.80 (0.58,1.11) | Alteplase  0.9 mg/kg | 0.72 (0.28,1.81) | 1.02 (0.81,1.28) | 0.81 (0.25,2.64) | 1.17 (0.67,2.06) | 1.04 (0.24,4.50) | 1.19 (0.68,2.07) | 1.05 (0.64,1.71) | 0.94 (0.21,4.19) | 0.68 (0.34,1.36) | 1.18 (0.76,1.82) |
| 1.12 (0.42,3.00) | 1.40 (0.55,3.55) | Tenecteplase 0.1 mg/kg | 1.42 (0.55,3.66) | 1.14 (0.34,3.80) | 1.64 (0.57,4.74) | 1.46 (0.26,8.25) | 1.66 (0.56,4.92) | 1.46 (0.51,4.18) | 1.31 (0.23,7.63) | 0.94 (0.30,3.02) | 1.64 (0.59,4.60) |
| 0.79 (0.53,1.17) | 0.98 (0.78,1.24) | 0.70 (0.27,1.81) | Tenecteplase 0.25 mg/kg | 0.80 (0.24,2.62) | 1.15 (0.67,1.99) | 1.02 (0.23,4.51) | 1.17 (0.63,2.16) | 1.03 (0.62,1.72) | 0.92 (0.20,4.16) | 0.66 (0.32,1.38) | 1.16 (0.71,1.88) |
| 0.98 (0.29,3.33) | 1.23 (0.38,3.98) | 0.88 (0.26,2.93) | 1.25 (0.38,4.09) | Tenecteplase 0.32 mg/kg | 1.44 (0.40,5.23) | 1.28 (0.20,8.36) | 1.46 (0.40,5.37) | 1.28 (0.36,4.57) | 1.15 (0.17,7.70) | 0.83 (0.21,3.25) | 1.44 (0.41,5.06) |
| 0.68 (0.36,1.31) | 0.85 (0.49,1.50) | 0.61 (0.21,1.76) | 0.87 (0.50,1.50) | 0.69 (0.19,2.52) | Tenecteplase 0.40 mg/kg | 0.89 (0.19,4.26) | 1.01 (0.46,2.24) | 0.89 (0.43,1.86) | 0.80 (0.16,3.94) | 0.58 (0.23,1.41) | 1.00 (0.49,2.04) |
| 0.77 (0.17,3.44) | 0.96 (0.22,4.14) | 0.69 (0.12,3.89) | 0.98 (0.22,4.30) | 0.78 (0.12,5.11) | 1.13 (0.23,5.40) | Reteplase 12+12 mg | 1.14 (0.26,4.91) | 1.00 (0.21,4.71) | 0.90 (0.11,7.30) | 0.65 (0.13,3.28) | 1.13 (0.25,5.19) |
| 0.68 (0.35,1.29) | 0.84 (0.48,1.47) | 0.60 (0.20,1.78) | 0.86 (0.46,1.58) | 0.69 (0.19,2.53) | 0.99 (0.45,2.19) | 0.88 (0.20,3.79) | Reteplase 18+18 mg | 0.88 (0.42,1.87) | 0.79 (0.16,3.91) | 0.57 (0.23,1.39) | 0.99 (0.49,2.01) |
| 0.77 (0.43,1.38) | 0.96 (0.59,1.56) | 0.68 (0.24,1.95) | 0.97 (0.58,1.62) | 0.78 (0.22,2.77) | 1.12 (0.54,2.34) | 1.00 (0.21,4.66) | 1.13 (0.54,2.40) | rhPro-UK 35mg | 0.90 (0.21,3.90) | 0.65 (0.28,1.51) | 1.12 (0.59,2.16) |
| 0.85 (0.19,3.95) | 1.07 (0.24,4.75) | 0.76 (0.13,4.43) | 1.08 (0.24,4.89) | 0.87 (0.13,5.81) | 1.25 (0.25,6.16) | 1.11 (0.14,9.00) | 1.27 (0.26,6.26) | 1.12 (0.26,4.85) | rhPro-UK 50mg | 0.72 (0.14,3.75) | 1.25 (0.26,5.95) |
| 1.19 (0.55,2.56) | 1.48 (0.74,2.97) | 1.06 (0.33,3.39) | 1.51 (0.72,3.14) | 1.21 (0.31,4.74) | 1.74 (0.71,4.26) | 1.54 (0.31,7.80) | 1.76 (0.72,4.29) | 1.55 (0.66,3.63) | 1.39 (0.27,7.23) | Staphylokinase 10mg | 1.74 (0.77,3.96) |
| 0.68 (0.40,1.17) | 0.85 (0.55,1.31) | 0.61 (0.22,1.70) | 0.86 (0.53,1.41) | 0.69 (0.20,2.43) | 1.00 (0.49,2.03) | 0.89 (0.19,4.07) | 1.01 (0.50,2.05) | 0.89 (0.46,1.71) | 0.80 (0.17,3.78) | 0.57 (0.25,1.31) | Placebo |

Abbreviation: rhPro-UK, Recombinant human prourokinase; Staphylokinase, Non-immunogenic recombinant staphylokinase

**Supplementary Table 14. Network sensitive-analysis of league diagram of symptomatic intracranial hemorrhage events.**

| Alteplase  0.6 mg/kg | 2.10 (1.17,3.76) | 3.19 (0.63,16.19) | 2.44 (1.27,4.69) | 2.97 (0.45,19.71) | 4.16 (1.53,11.26) | 6.70 (0.95,47.13) | 2.42 (0.89,6.57) | 1.13 (0.40,3.19) | 4.62 (0.32,66.10) | 0.77 (0.23,2.56) | 0.29 (0.07,1.10) |
| --- | --- | --- | --- | --- | --- | --- | --- | --- | --- | --- | --- |
| 0.48 (0.27,0.86) | Alteplase  0.9 mg/kg | 1.52 (0.33,6.93) | 1.16 (0.86,1.56) | 1.42 (0.23,8.57) | 1.98 (0.88,4.45) | 3.19 (0.50,20.57) | 1.16 (0.51,2.60) | 0.54 (0.23,1.27) | 2.20 (0.16,29.56) | 0.37 (0.13,1.05) | 0.14 (0.04,0.46) |
| 0.31 (0.06,1.59) | 0.66 (0.14,2.99) | Tenecteplase  0.1 mg/kg | 0.76 (0.17,3.51) | 0.93 (0.18,4.74) | 1.30 (0.25,6.88) | 2.10 (0.19,23.14) | 0.76 (0.14,4.23) | 0.35 (0.06,2.02) | 1.45 (0.07,29.24) | 0.24 (0.04,1.52) | 0.09 (0.01,0.63) |
| 0.41 (0.21,0.79) | 0.86 (0.64,1.16) | 1.31 (0.29,6.02) | Tenecteplase 0.25 mg/kg | 1.22 (0.20,7.45) | 1.71 (0.75,3.86) | 2.75 (0.42,18.11) | 0.99 (0.42,2.35) | 0.46 (0.19,1.15) | 1.90 (0.14,25.85) | 0.31 (0.11,0.94) | 0.12 (0.03,0.41) |
| 0.34 (0.05,2.23) | 0.71 (0.12,4.27) | 1.07 (0.21,5.47) | 0.82 (0.13,5.01) | Tenecteplase 0.32 mg/kg | 1.40 (0.20,9.77) | 2.25 (0.17,30.06) | 0.82 (0.11,5.87) | 0.38 (0.05,2.80) | 1.56 (0.07,36.64) | 0.26 (0.03,2.08) | 0.10 (0.01,0.85) |
| 0.24 (0.09,0.65) | 0.50 (0.22,1.13) | 0.77 (0.15,4.06) | 0.59 (0.26,1.33) | 0.71 (0.10,4.99) | Tenecteplase 0.40 mg/kg | 1.61 (0.21,12.26) | 0.58 (0.19,1.83) | 0.27 (0.08,0.88) | 1.11 (0.07,16.85) | 0.18 (0.05,0.70) | 0.07 (0.02,0.30) |
| 0.15 (0.02,1.05) | 0.31 (0.05,2.02) | 0.48 (0.04,5.26) | 0.36 (0.06,2.40) | 0.44 (0.03,5.91) | 0.62 (0.08,4.73) | Reteplase 12+12 mg | 0.36 (0.06,2.33) | 0.17 (0.02,1.31) | 0.69 (0.03,16.84) | 0.11 (0.01,0.97) | 0.04 (0.00,0.39) |
| 0.41 (0.15,1.12) | 0.87 (0.39,1.94) | 1.32 (0.24,7.34) | 1.01 (0.42,2.38) | 1.23 (0.17,8.83) | 1.72 (0.55,5.39) | 2.76 (0.43,17.79) | Reteplase 18+18 mg | 0.47 (0.14,1.52) | 1.91 (0.13,28.93) | 0.32 (0.08,1.20) | 0.12 (0.03,0.51) |
| 0.88 (0.31,2.49) | 1.85 (0.79,4.37) | 2.82 (0.49,16.09) | 2.15 (0.87,5.34) | 2.62 (0.36,19.28) | 3.67 (1.13,11.93) | 5.92 (0.76,45.97) | 2.14 (0.66,6.96) | rhPro-UK 35mg | 4.08 (0.30,54.75) | 0.68 (0.17,2.64) | 0.25 (0.06,1.12) |
| 0.22 (0.02,3.10) | 0.45 (0.03,6.09) | 0.69 (0.03,13.96) | 0.53 (0.04,7.20) | 0.64 (0.03,15.14) | 0.90 (0.06,13.65) | 1.45 (0.06,35.38) | 0.52 (0.03,7.96) | 0.24 (0.02,3.29) | rhPro-UK 50mg | 0.17 (0.01,2.73) | 0.06 (0.00,1.09) |
| 1.30 (0.39,4.35) | 2.73 (0.95,7.85) | 4.16 (0.66,26.38) | 3.18 (1.06,9.51) | 3.87 (0.48,31.21) | 5.42 (1.44,20.47) | 8.74 (1.03,74.25) | 3.16 (0.84,11.94) | 1.48 (0.38,5.75) | 6.03 (0.37,99.31) | Staphylokinase 10mg | 0.37 (0.07,1.87) |
| 3.50 (0.91,13.54) | 7.35 (2.17,24.87) | 11.19 (1.60,78.23) | 8.54 (2.43,29.96) | 10.41 (1.18,91.55) | 14.57 (3.37,62.90) | 23.47 (2.53,217.35) | 8.49 (1.96,36.69) | 3.97 (0.89,17.61) | 16.19 (0.92,285.01) | 2.69 (0.54,13.47) | Placebo |

Abbreviation: rhPro-UK, Recombinant human prourokinase; Staphylokinase, Non-immunogenic recombinant staphylokinase

**Supplementary Table 15. Network sensitive-analysis of rank of different dose of thrombolytics on excellent functional outcome at 90 days.**

| **Treatment** | **SUCRA** | **PrBest** | **MeanRank** |
| --- | --- | --- | --- |
| Alteplase 0.6 mg/kg | 26.7 | 0.0 | 9.1 |
| Alteplase 0.9 mg/kg | 39.7 | 0.0 | 7.6 |
| Tenecteplase 0.1 mg/kg | 34.8 | 0.1 | 8.2 |
| Tenecteplase 0.25 mg/kg | 55.9 | 0.0 | 5.8 |
| Tenecteplase 0.32 mg/kg | 41.6 | 1.2 | 7.4 |
| Tenecteplase 0.40 mg/kg | 50.6 | 0.1 | 6.4 |
| Reteplase 12+12 mg | 37.0 | 1.0 | 7.9 |
| Reteplase 18+18 mg | 87.5 | 7.1 | 2.4 |
| rhPro-UK 35 mg | 60.8 | 0.0 | 5.3 |
| rhPro-UK 50 mg | 60.3 | 8.8 | 5.4 |
| Staphylokinase 10 mg | 97.8 | 81.7 | 1.2 |
| Placebo | 7.1 | 0.0 | 11.2 |

Abbreviation: SUCRA, surface under the cumulative ranking; PrBest, probability of being the best; rhPro-UK, Recombinant human prourokinase; Staphylokinase, Non-immunogenic recombinant staphylokinase

**Supplementary Table 16. Network sensitive-analysis of rank of different dose of thrombolytics on good functional outcome at 90 days.**

| **Treatment** | **SUCRA** | **PrBest** | **MeanRank** |
| --- | --- | --- | --- |
| Alteplase 0.6 mg | 52.4 | 3.5 | 6.2 |
| Alteplase 0.9 mg | 56.3 | 0.0 | 5.8 |
| Tenecteplase 0.1 mg | 34.2 | 4.5 | 8.2 |
| Tenecteplase 0.25 mg | 56.3 | 0.5 | 5.8 |
| Tenecteplase 0.32 mg | 34.8 | 5.5 | 8.2 |
| Tenecteplase 0.40 mg | 65.8 | 20.2 | 4.8 |
| Reteplase 12+12 mg | 28.3 | 3.1 | 8.9 |
| Reteplase 18+18 mg | 87.6 | 44.2 | 2.4 |
| rhPro-UK 35mg | 58.2 | 3.2 | 5.6 |
| rhPro-UK 50mg | 35.7 | 8.1 | 8.1 |
| Staphylokinase 10mg | 51.5 | 3.2 | 6.3 |
| Placebo | 38.9 | 4.0 | 7.7 |

Abbreviation: SUCRA, surface under the cumulative ranking; PrBest, probability of being the best; rhPro-UK, Recombinant human prourokinase; Staphylokinase, Non-immunogenic recombinant staphylokinase

**Supplementary Table 17. Network sensitive-analysis of rank of different dose of thrombolytics on 90-day all-cause mortality events.**

| **Treatment** | **SUCRA** | **PrBest** | **MeanRank** |
| --- | --- | --- | --- |
| Alteplase 0.6 mg/kg | 29.6 | 0.1 | 8.7 |
| Alteplase 0.9 mg/kg | 53.0 | 0.1 | 6.2 |
| Tenecteplase 0.1 mg/kg | 30.1 | 3.1 | 8.7 |
| Tenecteplase 0.25 mg/kg | 55.1 | 0.5 | 5.9 |
| Tenecteplase 0.32 mg/kg | 41.0 | 9.8 | 7.5 |
| Tenecteplase 0.40 mg/kg | 67.8 | 12.5 | 4.5 |
| Reteplase 12+12 mg | 55.2 | 25.4 | 5.9 |
| Reteplase 18+18 mg | 68.6 | 12.4 | 4.5 |
| rhPro-UK 35 mg | 57.6 | 4.4 | 5.7 |
| rhPro-UK 50 mg | 48.7 | 20.9 | 6.6 |
| Staphylokinase 10 mg | 23.3 | 0.9 | 9.4 |
| Placebo | 69.9 | 9.9 | 4.3 |

Abbreviation: SUCRA, surface under the cumulative ranking; PrBest, probability of being the best; rhPro-UK, Recombinant human prourokinase; Staphylokinase, Non-immunogenic recombinant staphylokinase

**Supplementary Table 18. Network sensitive-analysis of rank of of different dose of thrombolytics on symptomatic intracranial hemorrhage events.**

| **Treatment** | **SUCRA** | **PrBest** | **MeanRank** |
| --- | --- | --- | --- |
| Alteplase 0.6 mg/kg | 22.5 | 0.0 | 9.5 |
| Alteplase 0.9 mg/kg | 49.8 | 0.0 | 6.5 |
| Tenecteplase 0.1 mg/kg | 66 | 7.9 | 4.7 |
| Tenecteplase 0.25 mg/kg | 60.2 | 0.1 | 5.4 |
| Tenecteplase 0.32 mg/kg | 63.0 | 9.8 | 5.1 |
| Tenecteplase 0.40 mg/kg | 79.2 | 9.9 | 3.3 |
| Reteplase 12+12 mg | 83.9 | 40.5 | 2.8 |
| Reteplase 18+18 mg | 57.8 | 0.8 | 5.6 |
| rhPro-UK 35 mg | 27.3 | 0.0 | 9.0 |
| rhPro-UK 50 mg | 71.0 | 30.9 | 4.2 |
| Staphylokinase 10 mg | 17.2 | 0.0 | 10.1 |
| Placebo | 2.3 | 0.0 | 11.7 |

Abbreviation: SUCRA, surface under the cumulative ranking; PrBest, probability of being the best; rhPro-UK, Recombinant human prourokinase; Staphylokinase, Non-immunogenic recombinant staphylokinase
